# Analytical Method Development for 19 Alkyl Halides as Potential Genotoxic Impurities by Analytical Quality by Design

**DOI:** 10.3390/molecules27144437

**Published:** 2022-07-11

**Authors:** Kyoungmin Lee, Wokchul Yoo, Jin Hyun Jeong

**Affiliations:** 1 College of Pharmacy, Yonsei Institute of Pharmaceutical Sciences, Yonsei University, 85 Songdogwahak-ro, Yeonsu-gu, Incheon 21983, Korea; lkm@hanmi.co.kr (K.L.); panda8838@hanmi.co.kr (W.Y.); 2 Analytical Research Department, Central Research Institute, Hanmi fine Chemical, 59 Gyeongje-ro, Siheung-si 15093, Korea

**Keywords:** GC–MS, analytical QbD, genotoxic impurity, alkyl halide, (Q)SAR, analytical method development

## Abstract

Major issues in the pharmaceutical industry involve efficient risk management and control strategies of potential genotoxic impurities (PGIs). As a result, the development of an appropriate method to control these impurities is required. An optimally sensitive and simultaneous analytical method using gas chromatography with a mass spectrometry detector (GC–MS) was developed for 19 alkyl halides determined to be PGIs. These 19 alkyl halides were selected from 144 alkyl halides through an in silico study utilizing quantitative structure–activity relationship (Q-SAR) approaches via expert knowledge rule-based software and statistical-based software. The analytical quality by design (QbD) approach was adopted for the development of a sensitive and robust analytical method for PGIs. A limited number of literature studies have reviewed the analytical QbD approach in the PGI method development using GC–MS as the analytical instrument. A GC equipped with a single quadrupole mass spectrometry detector (MSD) and VF-624 ms capillary column was used. The developed method was validated in terms of specificity, the limit of detection, quantitation, linearity, accuracy, and precision, according to the ICH Q2 guideline.

## 1. Introduction

Alkyl halides, also known as haloalkanes or halogenoalkanes, are chemical compounds formed from alkanes containing one or more halogen atoms, such as chlorine, bromine, fluorine, or iodine. These compounds are mainly used in alkylation reactions via nucleophilic substitutions as starting materials or reagents in synthetic processes of active pharmaceutical ingredients (APIs) because of their good reactivity, ease of use, reasonable prices, and commercial availability. Alkyl halides are considered potential genotoxic impurities (PGIs) because they can possibly alkylate DNA bases (on N-7 of guanine and N-3 of adenine) [1,2,3,4]. Alkyl halides are potentially formed during API manufacturing processes during undesired chemical reactions or are carried over to APIs when used as starting materials or reagents. For these reasons, alkyl halides should be considered significant PGIs when developing manufacturing processes and designating quality control strategies for APIs to manage genotoxic impurities [5,6,7,8]. 

The concept of structural alerts for genotoxicity was established by Ashby and Tennant in the 1980s [9]. As a result of this establishment, mutagenic and genotoxic in vitro and in vivo studies for many substances have been conducted. However, these experiments had limitations (in that the results could not be obtained for all substances because of the costs and time requirements). In particular, there is a lack of genotoxic evaluation data for alkyl halides commonly used in the pharmaceutical industry. Additional studies and experiments are being conducted to mitigate these limitations. Regulatory authorities, such as the FDA and EMEA, have also expanded their compilations of genotoxicity research data and are continuously publishing guidelines for the pharmaceutical industry as addendums. In silico studies using quantitative structure–activity relationship (Q-SAR) prediction programs could be effective alternative approaches in terms of cost- and time-saving. In many studies, Q-SAR prediction models showed excellent predictive performances and could serve as early warning systems for the prediction of genotoxicity in compounds. 

Once the compounds are confirmed to have potential genotoxicity through the use of Q-SAR prediction programs, the manufacturing processes of APIs should be altered to remove or minimize these substances classified as PGIs. However, complete elimination is often not possible; in this case, the amount of PGIs present should be limited to specific levels because of their genetically-threatening behaviors. PGIs are controlled according to EMEA and FDA guidelines because of the different risks and behaviors they impose, unlike other general impurities that are controlled according to the ICH Q3A and Q3B guidelines. The limits of PGIs are established based on the experimental results of toxicities that may be applied according to the threshold of toxicological concern (TTC), which is set at 1.5 µg/day. Generally, the limits of PGIs are much lower than the typical limit of non-PGIs, as even a trace amount of PGIs can affect drug quality and human health. Therefore, it is essential to develop a highly sensitive analytical method capable of detecting limits of sub-ppm for potential genotoxic substances for manufacturing process research and product quality control. Several analytical methods have already been developed with high sensitivity for PGIs and alkyl halides to meet these requirements in other previous literature studies [10,11,12,13,14]. Most of these analysis methods were performed using a gas chromatography or liquid chromatography system, altered according to the characteristics of each impurity, alongside a mass spectrometry detector capable of detecting trace amounts of impurities. Other literature studies used detectors other than the mass spectrometry detector along with their respective chromatographic systems [15,16,17,18,19,20,21]. The analytical quality by design (QbD) approach can be a good tool for the development of an optimally robust analytical method for PGIs [22,23]. The analytical QbD is a systematic approach that applies the QbD concept to the analytical method development to facilitate regulatory flexibility and prevent the undesired risks of quality control, such as out of specification (OOS) and out of trend (OOT), by increasing the scientific understanding of analytical methods.

Although the genotoxic behaviors of alkyl halides are widely known, experimental mutagenicity and/or carcinogenicity results, as well as Q-SAR analysis results, are limited. control strategies based on risk assessment and classification according to the ICH M7 guidelines are required because alkyl halides are essential compounds in the pharmaceutical industry [24]. Applying the analytical QbD approach can be effective in developing an optimally robust analytical method, which is one of the most essential procedures in the control strategies for PGIs. Many researchers have adopted the analytical QbD principle in the development of analytical methods of APIs according to numerous literature studies [25,26,27,28,29,30,31,32,33,34]. However, the majority of these research studies focused on the purity and assay method development of non-PGIs. In particular, few literature studies have applied the analytical QbD approach to the development of analytical methods using gas chromatography as the source of instrumentation, which is an effective analytical method for volatile substances, such as alkyl halides. 

**Figure 1:**
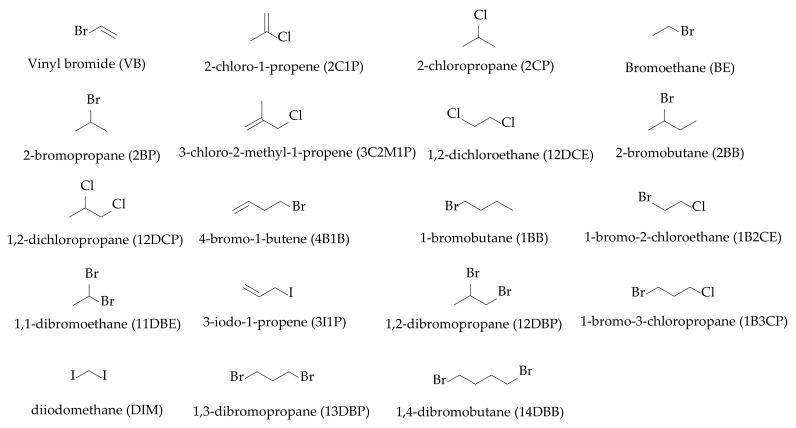
Chemical structures and abbreviations of the 19 selected alkyl halides.

In this study, an in silico experiment was performed using the Q-SAR approach for 144 alkyl halides with one to four carbons and one to two halogen atoms. As a result of the in silico experiment, the alkyl halides were classified from one to five according to the ICH M7 guideline. A total of 19 alkyl halides were selected as having the highest risks among the 144 alkyl halides evaluated through the risk assessment (Figure 1). The analytical method development using the analytical QbD approach and GC–MS focused on high sensitivity, specificity, and reproducibility for the selected 19 alkyl halides. In most literature studies about the analytical method development employing the analytical QbD approach, the resolution and analysis time were selected as critical method attributes (CMAs). In this study, the peak area and resolution of each peak were selected as the CMAs due to the most important analytical target profiles (ATPs) in the analytical method for PGIs being detection sensitivity and specificity. In addition, the analytical method validation was conducted according to the ICH Q2 guidelines in terms of system suitability, specificity, linearity, range, accuracy, precision, and robustness [35].

## 2. Results and Discussion

### 2.1. In Silico Study for PGIs

Alkyl halides are chemicals widely known to be genotoxic. Alkyl halides consisting of one to four carbons were selected as PGIs for the in silico study because these alkyl halides have a high risk of genotoxicity due to their relatively small sizes. These alkyl halides also contain one or two halogens and/or oxygen atoms. After selecting a total of 144 alkyl halides, risk assessments using in silico programs were performed according to the ICH M7 guidelines. Derek Nexus, which is an expert, knowledge-based software, and Sarah Nexus, which is a statistical-based software, were applied complementary to one another to evaluate genotoxicity. Since the prediction results between the two programs may be the same or contradict one another, the results were carefully evaluated. Derek Nexus and Sarah Nexus were both developed by the same company called LHASA and run on the same interface. Since PGIs are automatically classified according to the ICH M7 guidelines (through the complementary combination of the two results from Derek and Sarah), there is an advantage in that the personal opinions of researchers on the classifications of the target analytes could be minimized. Both Derek Nexus and Sarah Nexus were properly validated according to OECD validation principles. VEGA Consensus, a free program, was used to verify the results of both programs.

From the results of the ICH M7 classification, it was found that among a total of 144 alkyl halides, 11 alkyl halides were known mutagenic carcinogens in class 1, 31 alkyl halides were known mutagens with unknown carcinogenic potential in class 2, 82 alkyl halides had alerting structures and no mutagenicity data in class 3, and 16 alkyl halides had no structural alerts or sufficient data to demonstrate a lack of mutagenicity or carcinogenicity in class 5. Only four alkyl halides were classified as inconclusive. Among the 144 alkyl halides, 124 alkyl halides were classified as classes 1, 2, and 3, which could be considered potential genotoxic impurities, and only 16 alkyl halides were classified as class 5, which could be considered non-genotoxic impurities. The Derek and Sarah prediction results showed minor differences from one another.

According to the literature review, many experimental studies, such as the Ames test, have been conducted on alkyl halides in which positive results were obtained in many cases [36,37,38,39,40,41]. 

Most alkyl halides can be considered hazardous and extra precautions must be taken when they are incorporated into any API manufacturing process. However, it is difficult to avoid the use of alkyl halides in API manufacturing processes. Therefore, it is essential to establish manufacturing processes that can reliably remove residual alkyl halides and develop a quality control strategy for these alkyl halides. The quantitative toxicity results for only seven alkyl halides were obtained from a literature review [42] and it was concluded that there were no quantitative toxicity results for the other 137 alkyl halides. Since there are no studies on the quantitative toxicities of most alkyl halides, alkyl halide quantification was controlled using conventional limits by the TTC concept to define an acceptable intake according to the ICH M7 guidelines.

For the selection of the target alkyl halides for this study, 144 compounds were classified from classes 1 to 5 through an in silico study. Among the compounds classified as classes 1 and 2, 19 target alkyl halides were selected based on the availability of the reference standards of these alkyl halides in South Korea. According to the ICH M7 guidelines, compounds in classes 1 and 2 must be heavily regulated as compounds in class 1 are known mutagenic carcinogens, and compounds in class 2 are known mutagens with unknown carcinogenic potential. The genotoxicity predictions for 19 selected alkyl halides are shown in Table 1.

### 2.2. Analytical Method Development by Analytical QbD

**Figure 2:**
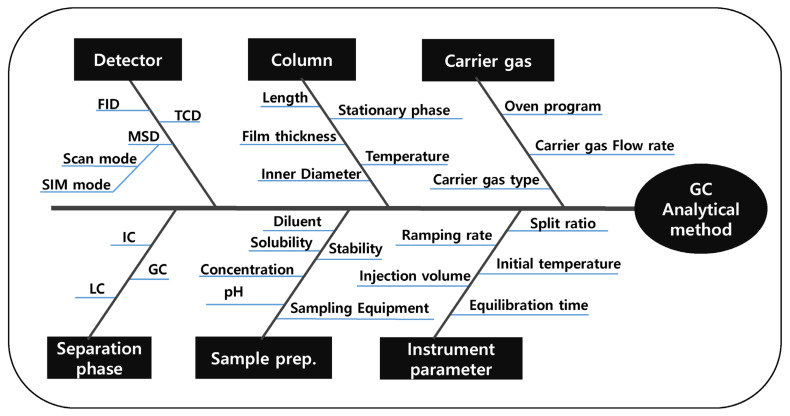
Fishbone diagram.

An analytical method for PGIs should have good detection sensitivity to control PGIs with low acceptance criteria. Moreover, each peak should be well separated and not interfere with each other to analyze multiple PGIs simultaneously. For this reason, the ATP was set to achieve optimal detection sensitivity and separation between adjacent peaks. According to the established ATPs, CMAs were set as the peak areas (to increase sensitivity as much as possible) and the resolution (to secure sufficient separation between each peak). The potential critical method parameters (CMPs) selected were the initial oven temperature, oven temperature hold time, oven temperature ramping rate, column flow rate, sample injection volume, final oven temperature, type of carrier gas, sample injector temperature, detector temperature, type of column, sample injection route, type of detector, and type of diluent. These parameters were selected from the fishbone diagram and the initial risk assessment based on a cause and effect (C&E) analysis. The experimental strategy for each parameter was classified under one-factor-at-a-time (OFAT), fixed, controlled, or design of experiment (DoE), based on the risk assessment performed using the failure mode and effect analysis (FMEA). The parameters classified under OFAT in the experimental strategy, such as column type, injection type, type of detector, and diluent were determined through the method scouting process. The effects on the results of the parameters, such as flow rate, initial temperature, ramping rate, and injector temperature, which are classified under DoE in the experimental strategy, were confirmed through the method screening process. The fishbone diagram is shown in Figure 2. 

#### 2.2.1. Method Scouting

The analytical method scouting was conducted by multiple OFAT experiments. The types of columns, injection equipment, detectors, and diluents were screened. Proper selection of the dilution solvent must be carried out to ensure complete solubility of all the target analytes and prevent other side reactions that can occur during analysis. When choosing the injection method, the boiling point of the target analytes must be considered, which is an important parameter that can particularly influence the detection sensitivity. The two common injection methods in gas chromatography are the auto-sampler method (in which the liquid analyte is directly injected) and the headspace method (in which the analyte is injected after vaporization). The type of column is a factor that has an overall effect on the results of gas chromatography. It mainly affects THE retention time, sensitivity, and resolution of THE analytes. The type of detector is also an important parameter; to find better reproducibility and sensitivity, a comparative test between the flame ionization detector (FID) and mass spectrometer detector (MSD) was conducted. VF-624 (6% cyanopropylphenyl/94% dimethylpolysiloxane) and DB-WAX (100% polyethylene glycol) capillary columns with different packing materials were tested by OFAT experiments; VF-624 (with the best resolution) was selected. In the case of DB-wax, the column did not have sufficient retention, all impurities were detected before 10 min, and sufficient separation was not achieved.

The split ratio of 2:1 was chosen for the split mode because the splitless injection method caused overloading of peaks that disrupted the separation efficiency of the column, and sufficient sensitivity was not achieved at a split ratio greater than 2:1. To select an appropriate detector, the FID and MSD, which are generally used in gas chromatography, were compared. The FID is known to have better reproducibility and is more commonly used in pharmaceutical analyses. However, the FID did not produce the sufficient sensitivity needed to quantify trace amounts of PGIs, which are controlled at low limits using the concept of TTC. Therefore, the MSD was chosen as the detector using the selected ion monitoring (SIM) mode to maintain optimal sensitivity. The dilution solvent was selected based on its non-reactivity with the target compound and solubility. The chosen dilution solvent must also not interfere with the peak of the target impurities in the chromatograph. The selection process of the dilution solvent was carried out with dimethyl sulfoxide, dimethyl formamide, methanol, acetonitrile, purified water, dimethyl acetamide, and dichloromethane, which are commonly used in gas chromatography as dilution solvents. Because of their differences in solubility, all of the target alkyl halides were unable to dissolve in the same dilution solvents at high concentrations. Therefore, the analysis method was separated into methods A and B, with 13 alkyl halides dissolved in dimethyl sulfoxide, and 6 alkyl halides dissolved in acetonitrile, respectively. 

#### 2.2.2. Method Screening

**Figure 3:**
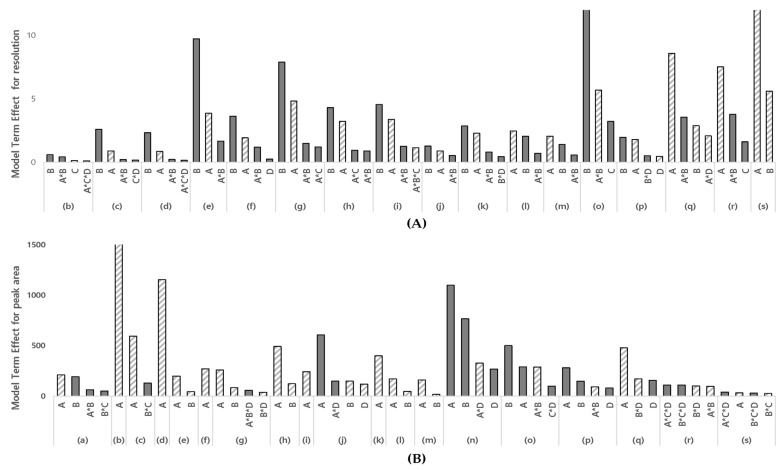
Model terms ranking the Pareto charts, depicting the influences of the initial oven temp., the flow rate of the carrier gas, the oven temp. ramping rate, and the sample injector temp. (**A**) Pareto chart for resolution; (**B**) Pareto chart for peak area; (**A**) initial oven temp., (**B**) the flow rate of the carrier, C oven temp. ramping rate D sample injector temp; (a) VB, (b) 2C1P, (c) 2CP, (d) BE, (e) 2BP, (f) 3C2M1P, (g) 12DCE, (h) 2BB, (i) 12DCP, (j) 4B1B, (k) 1BB, (l) 1B2CE, (m) 11DBE, (n) 3I1P, (o) 12DBP, (p) 1B3CP, (q) DIM, (r) 13DBP, (s) 14DBB. The height of the bar is the magnitude of the corresponding model term’s effect on the response. A dashed bar corresponds to a positive effect, while a solid bar corresponds to a negative effect, and the interaction between parameters is marked with an asterisk (*).

Method screening was conducted to identify the significant parameters and interactions of the parameters critically affecting the pre-selected CMAs. The initial oven temperature, flow rate of carrier gas in column, oven temperature ramping rate, and sample injector temperature were selected as the CMPs through the method scouting process and risk assessment. Using Fusion QbD software, a DoE with a two-level-full factorial design with four center points was designed and tested with the CMPs that are expected to have critical effects on the selected response, resolution, and peak area. The DoE is tabulated in Table 2. A regression analysis of variance analysis (ANOVA) was evaluated to check the appropriate model fitting (Table 3). The statistical significance was confirmed using the F-ratio and *p*-value for each PGI analyzed. The calculated Pareto charts were reviewed to quantitatively identify the effects and interactions of each parameter on the established CMAs. The model terms ranking the Pareto charts are summarized in Figure 3. The Pareto charts show that the flow rate, initial temperature, or both parameters had the greatest effects on the resolution and peak area except for the curvature effect. A blue-colored bar corresponds to a positive effect, while a gray-colored bar corresponds to a negative effect. The flow rate was shown to have a positive effect on the peak area, while the initial temperature had a negative effect on the resolution. The selected responses were not affected or partially affected by the ramping rate of the oven and sample injector temperature. As a result, the flow rate of the carrier gas and the initial temperature of the oven were selected as the CMPs for the method optimization study.

#### 2.2.3. Method Optimization by Analytical QbD

The response surface methodology (RSM), which is based on standard orthogonal arrays that contain three levels for each experiment variable, was employed for the method optimization study. To meet the goal of the analytical method optimization, the model can provide data from which linear and curvilinear variable behaviors can be quantified within the allowable ranges. The critical parameters should be properly selected for optimization because optimization studies require a relatively large number of experiments compared to screening studies. Based on the results obtained from the method screening, the flow rate of the carrier gas in the column and the initial temperature of the column were selected as the CMPs for method optimization studies. Although it was not possible in the method screening process, the RSM model could provide information on curvature effects, which help to understand the correlation between various parameters. The DoE is tabulated in Table 4. A regression ANOVA was evaluated to check the appropriate model fitting (Table 5). The regression ANOVA statistics result was shown to be significant with a *p*-value and F-ratio for all responses.

**Figure 4:**
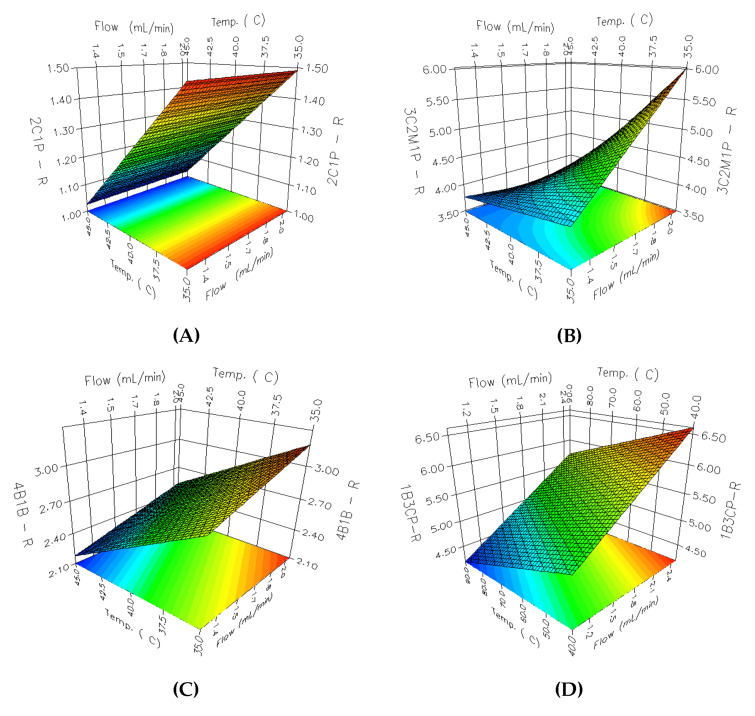
The 3D response surface plots for the resolution: (**A**) 2C1P; (**B**) 3C2M1P; (**C**) 4B1B; (**D**) 1B3CP.

**Figure 5:**
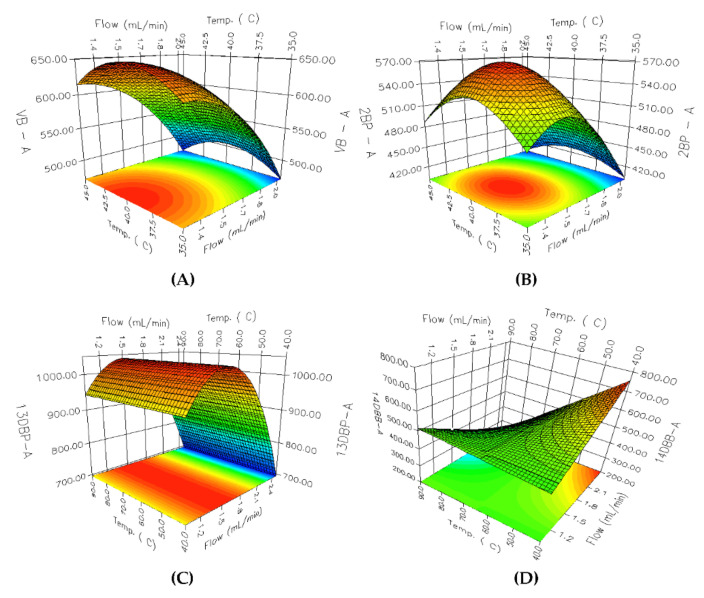
The 3D response surface plots for the peak area: (**A**) VB; (**B**) 2BP; (**C**) 13DBP; (**D**) 14DBB.

An RSM analysis was carried out employing 3D-response surface plots for identifying the underlying interaction among the selected parameters. A total of 36 3D response surface plots were obtained; among them, 8 PGIs with relatively low resolutions and peak areas were selected. Considering the parameter effects on PGIs with low resolutions and peak areas has a critical impact on the overall analytical method development. As shown in Figure 4, the effects of the flow rate and oven temperature on the resolution are different for each PGI. In the case of Figure 4A 2C1P, the resolution increases as the oven temperature decreases, whereas the increase in the flow rate of the carrier gas revealed negligible influence on the resolution. In the case of Figure 4A,C,D, a linear declining trend was observed for the resolution with an increase in the flow rate and a decrease in oven temperature. The response surface for Figure 4B 3C2M1P showed a curvilinear relationship between A and B, while the response surfaces for other PGIs showed linear relationships. The flow rate of the carrier gas and the oven temperature showed a more varied interaction with the change in the peak area. As shown in Figure 5, the peak area of Figure 5C 13DBP depends on the change of the flow rate and has a maximum point when the flow rate is 1.5 mL/min, while the effect of the column temperature on the peak area is negligible. Figure 5A VB, Figure 5B 2BP, and Figure 5D 14DBB portray considerably high levels of interaction between the flow rate of the carrier gas and the oven temperature for the peak area. Moreover, Figure 5A VB and Figure 5B 2BP produced a paraboloid that opened down and had a global maximum at its vertex within a given range, while Figure 5D 14DBB had a saddle point. 

**Figure 6:**
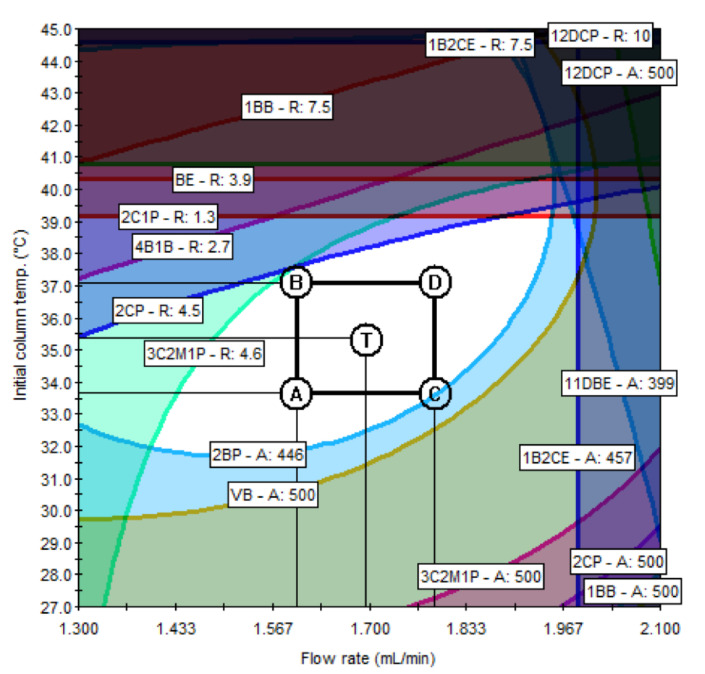
Method operable design region (MODR) and proven acceptable ranges (PARs) for method A.

**Figure 7:**
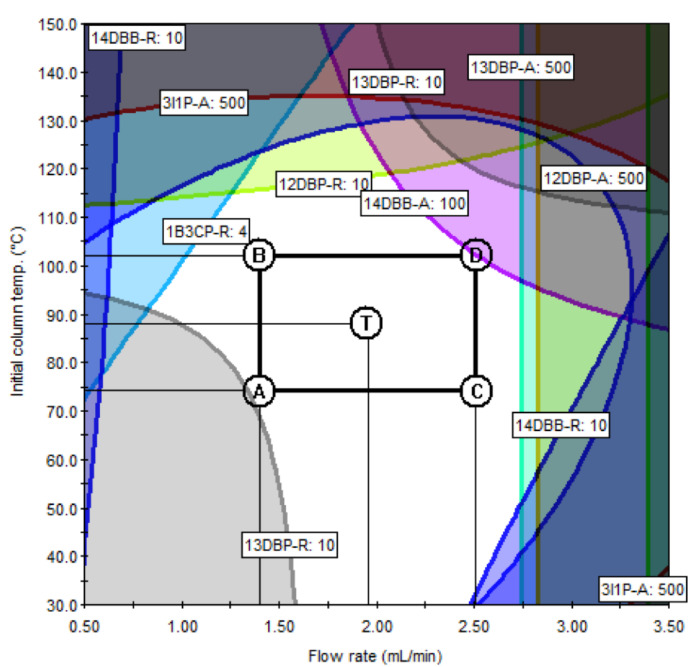
MODR and PARs for method B.

**Figure 8:**
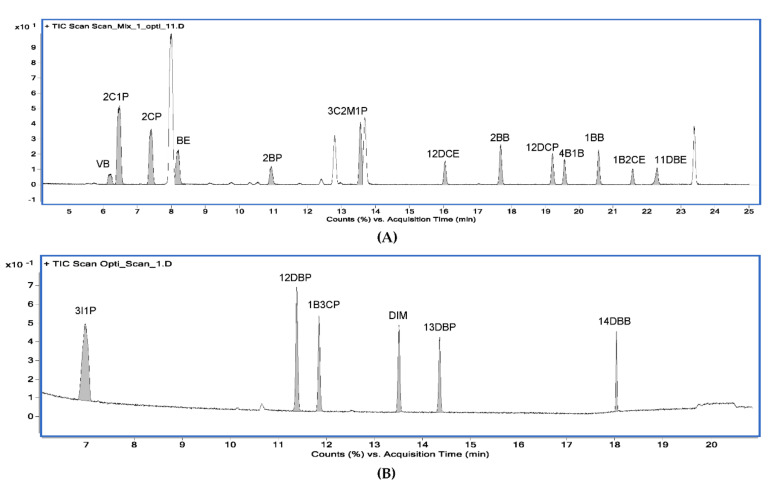
Typical GC−MS chromatograms of 19 alkyl halides using the scan mode. (**A**) Chromatogram for 13 alkyl halides obtained from method A; (**B**) chromatogram for 6 alkyl halides obtained from method B.

**Figure 9:**
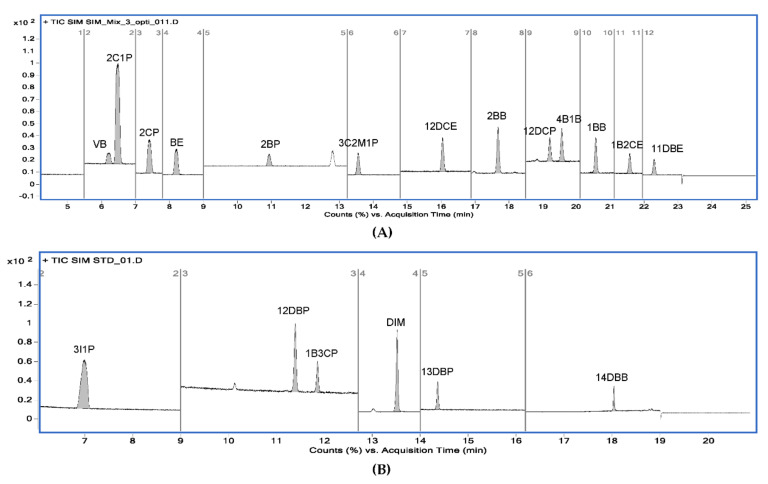
Typical GC−MS chromatograms of 19 alkyl halides using the selected ion monitoring (SIM) mode. (**A**) Chromatogram for 13 alkyl halides obtained from method A; (**B**) chromatogram for 6 alkyl halides obtained from method B.

According to the ICH Q14 guideline draft, the method operable design region (MODR) represents a combination of analytical procedure parameter ranges in which the analytical procedure performance criteria are fulfilled and the quality of the measured results is assured [43]. The optimum region was established by overlaying contours of all responses (each having an acceptance criterion). The acceptance criteria of the responses were set to satisfy the purposes of the ATPs. The acceptance criteria of the peak area were set at 500 or more to ensure sufficient sensitivity to detect trace amounts of PGIs, and the acceptance criteria of the resolution were set to 10 or greater so that each peak was sufficiently separated and there were no interferences with each other. If the predefined acceptance criteria were not met because of the unique chemical properties of certain PGIs, the acceptance criteria were set at 90% of the expected values. For the set acceptance criteria (10 for resolution and 500 for the peak area), the shaded contour represents the unacceptable region, while the unshaded represents the acceptable region. In the final optimization study, the unshaded region may represent the design space for the variables being studied in terms of the graphed response. The MODR in Figure 6 and Figure 7 show the regions for acceptable results for all responses, simultaneously. The proven acceptable range was defined inside of the predefined MODR, based on a linear combination of parameters, to the set center point of the parameter ranges. In the case of method A, the resolutions of 2CP, 3C2M1P, and 2C1P played important roles in determining the MODR. Since the sensitivity of 2BP is lower than that of other PGIs, the peak area of 2BP was confirmed as an important response. In the case of method B, the resolution of 1B3CP and the peak area of 14 DBB were important influences in determining the acceptable region. The typical GC−MS chromatograms using the scan mode and SIM mode are shown in Figure 8 and Figure 9, respectively.

### 2.3. Analytical Method Validation

The developed analytical method was validated according to the ICH Q2(R1) guidelines to demonstrate that the analytical procedure was suitable for its intended purpose. The analytical method was evaluated in the attributes of specificity, the limit of detection, the limit of quantitation, linearity, accuracy, and precision. A summary of the analytical method validation results is presented in Table 6. 

#### 2.3.1. Specificity

Specificity refers to the capability of the analytical method to selectively and accurately measure the target impurities in mixed states of impurities and/or degradants. Specificity was evaluated by the resolution between the adjacent peaks obtained from the standard solution. Specificity is a critical parameter in this method because multiple PGIs need to be analyzed simultaneously. The resolution between VB and 2C1P was determined to be the lowest with a resolution of 1.5. All of the resolution values between the adjacent peaks were above 1.5, which means that all peaks were well separated from each other for simultaneous analyses and the analytical procedure was proven to be specific for the target analytes. 

#### 2.3.2. Limit of Detection and Quantitation

The limit of detection (LOD) is the minimum detectable amount of each analyte present in the sample and the limit of quantitation (LOQ) is the minimum amount of each analyte to be analyzed among samples that could be expressed as quantitative values with appropriate precision. The test was conducted at a sufficiently low concentration to detect the signal-to-noise ratio of about 3 and 10 for the LOD and LOQ, respectively. The solutions with concentrations from about 0.5 to 0.8 ppm for each PGI were analyzed for the determinations of the LODs and LOQs. The calculated LODs for the PGIs were between 0.01 and 0.11 ppm and LOQs were between 0.03 and 0.38 ppm. The calculated LODs and LOQs were sufficiently low enough to control trace amounts of PGIs with low acceptance criteria.

#### 2.3.3. Linearity

Linearity of the test method refers to the ability of the analytical method to obtain a linear measurement value within a specified range in proportion to the amount (or concentration) of each analyte. Linearity was tested over the concentration range from the LOQ level to 2.4 ppm. Linearity was evaluated as a correlation coefficient (R), with all results being no less than 0.995; the proposed analytical method was proven to be linear for the target analytes. 

#### 2.3.4. Accuracy

Accuracy refers to the degree to which a measured value is close to a known true or standard value. Accuracy was evaluated by the recovery study of a matrix API being spiked with a PGI standard. The spiked sample solutions were prepared in triplicates at three concentration levels with the addition of known amounts of each PGI standard to the chosen API matrix. Raloxifene (Cas No. 84449-90-1) was used as a matrix API because alkyl halides are used in the manufacturing process of Raloxifene. The analyte contents in the spiked sample solutions were determined using the proposed analytical procedure and the recovery was calculated for each solution. All results met the acceptance criteria, within 100 ± 15% of recoveries for all PGIs, and the proposed analytical method was proven to be accurate for all target analytes. 

#### 2.3.5. Precision

Precision refers to the proximity between each measured value by taking multiple samples from a homogeneous sample and analyzing them according to the specified conditions. Precision was evaluated by the %RSD of the peak areas from six replicate injections of the LOQ solution (LOQ level precision) with a concentration at the LOQ level, and the standard solution (repeatability) with a concentration of 2 ppm. %RSD at the LOQ level was determined to be from 1.01 to 6.43% and %RSD at 2 ppm was determined to be from 1.94 to 6.60%, and the proposed analytical method was proven to be precise for all target analytes. 

### 2.4. Applicability of the Method to Real Sample

To ensure that the developed method applies to real APIs, raloxifene hydrochloride (Cas No. 82640-04-8), febuxostat (Cas No. 144060-53-7), sitagliptin phosphate (Cas No. 654671-78-0), amlodipine camsylate (Cas No. 652969-01-2), and ezetimibe (Cas No. 163222-33-1) were chosen and analyzed. All APIs were manufactured and supplied by Hanmi Fine Chemical in South Korea.

Since residual alkyl halides were not present in the chosen APIs, the matrix API samples were spiked with 19 of the alkyl halides at low concentrations.

Each test was repeated thrice, and the recovery, precision, specificity, LOD, and LOQ were analyzed each time using the matrix APIs. Recovery was determined to be between 86.0 and 102.7% for raloxifene hydrochloride, 77.9 and 110.2% for febuxostat, 77.0 and 106.4% for sitagliptin phosphate, 88.5 and 126.1% for amlodipine camsylate, and 81.8 and 117.6% for ezetimibe, respectively. 

From the analyses of matrix APIs using the proposed analytical method, it was confirmed that the matrix APIs do not interfere with the analyses of alkyl halides and the applicability of the method to real samples was confirmed with accuracy and precision. The detailed test results are tabulated in the Supplementary Materials.

## 3. Materials and Methods

### 3.1. Reagents, Materials, and Standards

The reference standards for vinyl bromide (Cas No. 593-60-2, purity: 98%), 2-chloro-1-propene (Cas No. 557-98-2, purity: 98%), 2-chloropropane (Cas No. 75-29-6, purity: 98%), 1,2-dichloroethane (Cas No. 107-06-2, purity: 99.8%), 4-bromo-1-butene (Cas No. 5162-44-7, purity: 97%), 1,1-dibromoethane (Cas No. 557-91-5, purity: 98%), 3-iodo-1-propene (Cas No. 513-48-4, purity: 98%), and diiodomethane (Cas No. 75-11-6, purity: 99%) were purchased from Sigma-Aldrich (Burlington, NJ, USA). 

The reference standards for bromoethane (Cas No. 74-96-4, purity: 99.0%), 2-bromopropane (Cas No. 75-26-3, purity: 99.0%), 3-chloro-2-methyl-1-propene (Cas No. 563-47-3, purity: 98.0%), 2-bromobutane (Cas No. 78-76-2, purity: 98.0%), 1,2-dichloropropane (Cas No. 78-87-5, purity: 98.0%), 1-bromobutane (Cas No. 109-65-9, purity: 98.0%), 1-bromo-2-chloroethane (Cas No. 107-04-0, purity: 98.0%), 1,2-dibromopropane (Cas No. 78-75-1, purity: 98.0%), 1-bromo-3-chloropropane (Cas No. 109-70-6, purity: 99.0%), 1,3-dibromopropane (Cas No. 109-64-8, purity: 98.0%), and 1,4-dibromobutane (Cas No. 110-52-1, purity: 98.0%) were purchased from TCI (Tokyo, Japan).

For the dilution solvents, *N*,*N*-dimethylacetamide (Cas No. 127-19-5, purity: 99.9%), *N*,*N*-dimethyl sulfoxide (Cas No. 67-68-5, purity: 99.0%), and dimethylformamide (Cas No. 75-12-7, purity: 99.5%) were purchased from Junsei Chemical (Tokyo, Japan). Dichloromethane (Cas No. 75-09-2, purity: 99.9%) and acetonitrile (Cas No. 75-05-8, purity: 99.9%) were purchased from Fisher Scientific (Pittsburgh, PA, USA). Methanol (Cas No. 67-56-1, purity: 99.8%) was purchased from Sigma-Aldrich (Burlington, USA). Purified water was provided from the Milli-Q water purification system (Burlington, MA, USA). The raloxifene hydrochloride (Cas No. 82640-04-8), febuxostat (Cas No. 144060-53-7), sitagliptin phosphate (Cas No. 654671-78-0), amlodipine camsylate (Cas No. 652969-01-2), and ezetimibe (Cas No. 163222-33-1) were obtained from Hanmi Fine Chemical (Gyeonggi-do, Korea).

For the method scouting process, a VF-624 ms capillary column (60 m × 0.25 mm i.d. × 1.4 μm film thickness, part no: CP9103) and DB-WAX capillary column (60 m × 0.53 mm i.d. × 1.0 μm film thickness, part no: 125-7062) were purchased from Agilent Technologies (Santa Clara, CA, USA).

### 3.2. In Silico Study

The program used for the in silico study was Derek Nexus (v 6.1.1, build 7 July 2021) from Lhasa Limited (Leeds, UK), which is an expert knowledge rule-based software according to the application of alerts and reasoning rules that cover structural alerts for various toxicological endpoints. For precise mutagenicity predictions, Sarah Nexus (v.3.1.1 built on 7 July 2021), also from Lhasa Limited, was used, which is a statistical-based software tool capable of calculating precise predictions of mutagenicity [44]. The ICH guidelines state that computational toxicity assessments can be conducted using two complementary methodologies for Q-SAR predictions that predict the bacterial mutation assay. One methodology can be expert rule-based and another can be statistical-based [45].

Vega (V 1.1.5-b48, built on 29 March 2021), free software in the VEGA HUB (www.vegahub.eu, accessed on 03 May 2022), was used as in silico prediction software for results regarding reliability [46].

### 3.3. Preparation of Solutions

Regarding the standard solution preparation for method A—0.1 g of vinyl bromide, 2-chloro-1-propene, 2-chloropropane, bromoethane, 2-bromopropane, 3-chloro-2-methyl-1-propene, 1,2-dichloroethane, 2-bromobutane, 1,2-dichloropropane, 4-bromo-1-butene, 1-bromobutane, 1-bromo-2-chloroethane and 1,1-dibromoethane—each was accurately weighed and transferred to a 100 mL volumetric flask, dissolved, and diluted with dimethylsulfoxide to the volume. Moreover, 0.5 mL of this solution was transferred to a 50 mL volumetric flask and diluted with dimethylsulfoxide to the volume. A total of 0.5 mL of this solution was transferred to a 50 mL volumetric flask and diluted with dimethylsulfoxide to volume. A total of 2.0 mL of this solution was transferred to a 10 mL volumetric flask and diluted with dimethylsulfoxide to volume.

A sample solution preparation for method A (0.1 g of the test specimen, accurately weighed) was transferred to a 10 mL volumetric flask, and then dissolved and diluted with dimethylsulfoxide to volume.

Regarding the standard solution preparation for method B—0.1 g of 3-iodo-1-propene, 1,2-dibromopropane, 1-bromo-3-chloropropane, diiodomethane, 1,3-dibromopropane and 1,4-dibromobutane—each was each accurately weighed, transferred to a 100 mL volumetric flask, dissolved, and diluted with acetonitrile to the volume. Moreover, 1.0 mL of this solution was transferred to a 100 mL volumetric flask and diluted with acetonitrile to the volume. A total of 0.5 mL of this solution was transferred to a 100 mL volumetric flask and diluted with acetonitrile to volume.

Regarding the sample solution preparation for method B—0.1 g of the test specimen was accurately weighed, transferred to a 10 mL volumetric flask, and then dissolved and diluted with acetonitrile to volume.

### 3.4. Analytical Condition and Equipment

GC condition. An Agilent 7890B gas chromatography instrument equipped with a 5977A Single Quadrupole MSD and FID (Agilent Technologies, Santa Clara, CA, USA) was used for this study. The sample injection was performed using an Agilent G7697A Headspace sampler and an 7693 autosampler (Agilent Technologies, Santa Clara, CA, USA). For the instrument control, data acquisition, and processing, Mass Hunter Qualitative Analysis ver. B.07.00 (Agilent Technologies) and OpenLAB CDS ChemStation edition Rev. C.01.07.SR4 (Agilent Technologies) were used. 

The analyses of PGIs with MSD were performed using a VF-624 ms capillary column (60 m × 0.25 mm i.d. × 1.4 μm film thickness). The column oven temperature programming was as follows: Method A—the initial oven temperature was set at 35 °C, then ramped at 3 °C/min to 124 °C. Method B—the initial oven temperature was set at 90 °C, then ramped at 2 °C/min to 100 °C, then ramped at 5 °C/min to 130 °C, and then ramped at 10 °C/min to 160 °C. The flow rate of the helium carrier gas was set at 1.7~2.0 mL/min with a mode of constant flow. The split/splitless inlet (SSL) was used as the inlet type and the injection port was used in the split mode at a split ratio of 2:1. For the injection, a 4 mm ID straight inlet liner was used. The injection heater temperature was set at 280 °C.

The headspace sampler (HSS) and automatic liquid sampler (ALS), which are the most common GC sampling techniques, were reviewed. Since the target impurities are easily volatilized due to their relatively low boiling points, both the HSS and ALS were utilizable and showed excellent reproducibility. However, in the case of ALS, there was a problem with the sample being carried over in the gas chromatography system, so the HSS was selected as the sampling technique. The injection volume was set at 1000 µL as the gas phase using the headspace sampler.

MS condition. The mass spectrometer was operated in the SIM under high-efficiency EI source conditions. The *m*/*z* values of 55.1, 57.1, 61.9, 63.0, 76.0, 77.0, 77.9, 89.9, 105.9, 106.9, 108.0, 121.0. 122.0, 137.0 155.9. 168.0. 200.0, and 268.0 ions with 50 ms dwell times were selected for analysis. The source temperature was set at 230 °C, the quadrupole temperature was set at 150 °C, and the transfer line temperature was set at 280 °C. The gain factor was set at 1.0. 

### 3.5. Method Development by Analytical QbD

The first step of the analytical QbD is to define a set of ATPs according to the purpose and scope of the analysis. The most important purpose of this analytical method is to analyze various and trace amounts of PGIs at once, so specificity and detection sensitivity were the ATPs assessed. The ATP corresponds to the quality target product profiles (QTPP) in a product QbD approach. To satisfy the defined ATPs, the CMAs need to be properly selected. Resolution and detection sensitivity were the chosen CMAs for the analytical method development for the PGIs assessed. The CMAs are equivalent to the critical quality attributes (CQAs) in a product QbD approach.

Risk assessment (RA). The risk assessment can be conducted in various ways. The potential risks related to the analytical method development were prioritized using the FMEA method. Based on the risk assessment performed, the potential risks were grouped into CMPs and controlled parameters. 

Method scouting. The method scouting process consisted of preliminary tests performed based on the risk assessment and knowledge gained through scientific experience using the OFAT method. 

Method screening. Method screening using the DoE approach was performed for the parameters that were classified as having high risks through the risk assessment. The DoE was conducted using the Fusion QbD software in a two-level full factorial design with four center points using a quadratic design model. The significance of the DoE was verified using the ANOVA statistical method. An understanding of the main effects, interactions, and Pareto charts between CMPs can be obtained from the screening results.

Method optimization. Method optimization was conducted for the parameters, which are defined as CMPs based on the results from the method screening process. The DoE was conducted in a central composite full type with four center points using the RSM approach. The RSM approach is useful in understanding the correlation between various parameters and responses. The significance of the DoE was verified using the ANOVA statistical analysis. The 3D-response surface plots exhibiting the effects of the CMPs and MODR were obtained using Fusion QbD software. 

### 3.6. Method Validation

The proposed analytical method was validated according to the ICH Q2 (R1) guideline. The validation parameters of specificity, the limit of detection, quantitation, accuracy, precision, and linearity of the analytical method were evaluated. 

Specificity was evaluated based on the resolution between PGI peaks from the chromatograms. It was ensured that none of the blank peaks interfered with the peaks of interest for the standard and sample solutions (confirmed by visual inspection). The prepared standard solution was injected into the GC in six replicates. The resolution between peaks was assessed using the computerized data system after peak integration and analysis.

The LOD and LOQ were determined by calculating the signal-to-noise (S/N) ratio obtained from the comparison of the signal (height) of each of the analytes and noise in the given time range closest to the analyte. The S/N ratios of about 3 and 10 were considered acceptable for estimating the LOD and LOQ.

Accuracy was evaluated using the recovery study of spiked PGIs. By the addition of known amounts of PGIs to a single batch of the matrix sample, the accuracy solutions were prepared in triplicates at three levels of the nominal concentration, and the percent recoveries of the impurities were calculated, respectively.

Precision was evaluated to ensure that the proposed analytical procedure was able to achieve closeness in the data results between the series of measures from several injections of the standard solution over a short interval of time.

Linearity was evaluated by showing that test results were directly proportional to the concentrations of the analytes over the specific ranges from the reporting levels (=LOQ) of genotoxic impurities to 120% of the specifications. The results are reported as the slope, y-intercept of the linear regression line, and correlation coefficient obtained from the analysis of the linearity solutions at five concentration levels, including the specific range.

## 4. Conclusions

A sensitive and simultaneous analytical method using GC–MS was successfully developed employing the analytical QbD method for 19 alkyl halides as PGIs in APIs. This study emphasizes the usefulness and efficiency of the QbD approach implementation in the development of the analytical method using GC–MS to quantify PGIs, which require low detection limits. Moreover, 144 alkyl halides were classified according to the ICH M7 guidelines through an in silico study using an expert knowledge-based and statistical-based program. A total of 19 alkyl halides, which require a highly sensitive analytical method, were selected because of their high potential for genotoxicity. Based on these results, the optimized analytical conditions were developed using the design space for the range of parameters that could satisfy the predefined CMAs. The analytical method validation for the developed method was performed in terms of specificity, the limit of detection, the limit of quantitation, linearity, accuracy, and precision according to the ICH Q2 guidelines. The developed method was confirmed to be appropriate and validated for the analysis of trace amounts of the 19 target PGIs.

## Figures and Tables

**Table 1:** The genotoxicity predictions for 19 selected alkyl halides.

Abbreviation	Name	CAS No.	Derek Prediction	Sarah Prediction	VEGA Prediction	ICH M7 Class
1BB	1-Bromobutane	109-65-9	Plausible	Positive	Positive	Class 2
BE	Bromoethane	74-96-4	Plausible	Positive	Positive	Class 1
VB	Vinyl bromide	593-60-2	Probable	Positive	Positive	Class 1
2BP	2-Bromopropane	75-26-3	Plausible	Positive	Positive	Class 2
2BB	2-Bromobutane	78-76-2	Plausible	Positive	Positive	Class 2
4B1B	4-Bromo-1-butene	5162-44-7	Plausible	Positive	Positive	Class 2
2CP	2-Chloropropane	75-29-6	Plausible	Positive	Positive	Class 2
2C1P	2-Chloro-1-propene	557-98-2	Plausible	Positive	Positive	Class 2
3C2M1P	3-Chloro-2-methyl-1-propene	563-47-3	Plausible	Positive	Positive	Class 1
3I1P	3-Iodo-1-propene	513-48-4	Plausible	Positive	Positive	Class 2
1B2CE	1-Bromo-2-chloroethane	107-04-0	Plausible	Positive	Positive	Class 2
1B3CP	1-Bromo-3-chloropropane	109-70-6	Plausible	Positive	Positive	Class 2
12DCE	1,2-Dichloroethane	107-06-2	Plausible	Positive	Positive	Class 1
12DCP	1,2-Dichloropropane	78-87-5	Plausible	Positive	Positive	Class 1
13DBP	1,3-Dibromopropane	109-64-8	Plausible	Positive	Negative	Class 2
11DBE	1,1-Dibromoethane	557-91-5	Plausible	Positive	Negative	Class 2
12DBP	1,2-Dibromopropane	78-75-1	Plausible	Positive	Positive	Class 2
14DBB	1,4-Dibromobutane	110-52-1	Plausible	Positive	Positive	Class 2
DIM	Diiodomethane	75-11-6	Probable	Positive	Negative	Class 2

**Table 2:** Design of experiment (DoE) for screening.

DoE for Method A					DoE for Method B				
No. Run	Flow Rate	Initial Temp.	Ramping Rate	Injector Temp.	No. Run	Flow Rate	Initial Temp.	Ramping Rate	Injector Temp.
A-1	2.0	35	2.5	225	B-1	2.5	40	2.0	225
A-2	2.0	65	2.5	225	B-2	2.5	90	2.0	225
A-3	0.3	35	2.0	225	B-3	1.0	40	1.5	225
A-4	2.0	35	2.5	215	B-4	2.5	40	2.0	215
A-5	2.0	35	2.0	225	B-5	2.5	40	1.5	225
A-6	2.0	35	2.0	215	B-6	2.5	40	1.5	215
A-7	0.3	35	2.5	225	B-7	1.0	40	2.0	225
A-8	2.0	65	2.5	215	B-8	2.5	90	2.0	215
A-9	1.2	50	2.3	220	B-9	1.8	65	1.8	220
A-10	1.2	50	2.3	220	B-10	1.8	65	1.8	220
A-11	1.2	50	2.3	220	B-11	1.8	65	1.8	220
A-12	0.3	65	2.5	215	B-12	1.0	90	2.0	215
A-13	2.0	65	2.0	215	B-13	2.5	90	1.5	215
A-14	0.3	65	2.5	225	B-14	1.0	90	2.0	225
A-15	0.3	65	2.0	215	B-15	1.0	90	1.5	215
A-16	0.3	65	2.0	225	B-16	1.0	90	1.5	225
A-17	2.0	65	2.0	225	B-17	2.5	90	1.5	225
A-18	1.2	50	2.3	220	B-18	1.8	65	1.8	220
A-19	0.3	35	2.5	215	B-19	1.0	40	2.0	215
A-20	0.3	35	2.0	215	B-20	1.0	40	1.5	215

**Table 3:** Regression analysis of variance analysis (ANOVA) statistics results in the screening.

		Sum of Squares	DF*	Mean Square	F-Ratio	*p*-Value		Sum of Squares	DF	Mean Square	F-Ratio	*p*-Value
VB	A*	363,545	4	90,886	123.65	<0.01	3I1P	7,891,128	5	1,578,225	35.98	<0.01
2C1P	R*	2.31	9	0.25	115.00	<0.01	1BB	57.83	4	14.45	39.10	<0.01
	A	28,372,838	1	28,372,838	134.66	<0.01		703,483	1	703,483	84.49	<0.01
2CP	R	2.50	6	0.41	723.67	<0.01	1B2CE	44.15	3	14.71	58.83	<0.01
	A	1,617,766	2	808,883	55.08	<0.01		135,246	2	67,623	38.70	<0.01
BE	R	25.01	7	3.57	382.44	<0.01	11DBE	26.54	3	8.8497	62.24	<0.01
	A	5,578,546	1	5,578,546	171.11	<0.01		109,070	2	54,535	240.22	<0.01
2BP	R	449.97	3	149.99	839.29	<0.01	12DBP	5,359	3	1,786	176.64	<0.01
	A	174,573	2	87,286	58.73	<0.01		1,562,157	5	312,431	47.35	<0.01
3C2M1P	R	60.00	5	12.00	675.49	<0.01	1B3CP	31.74	10	3.17	122.00	<0.01
	A	313,435	1	313,435	133.11	<0.01		512,151	10	51,215	57.34	<0.01
12DCE	R	370.90	8	46.36	207.03	<0.01	DIM	308.94	6	51	133.18	<0.01
	A	336,637	6	56,106	185.06	<0.01		1,205,161	3	401,720	19.31	<0.01
2BB	R	127.72	7	18.24	114.85	<0.01	13DBP	1.63	3	0.54	79.58	<0.01
	A	1,122,779	2	561,389	56.16	<0.01		<0.0001	9	<0.0001	20.92	<0.01
12DCP	R	144.53	5	28.90	159.55	<0.01	14DBB	3,483	2	1,741	153.26	<0.01
	A	252,402	1	252,402	176.19	<0.01		12,128	7	1,732	8.42	<0.01
4B1B	R	10.91	3	3.63	66.17	<0.01						
	A	0.0003	4	<0.0001	36.66	<0.01						

A*: peak area, R*: resolution, DF*: degree of freedom.

**Table 4:** DoE for optimization.

DoE for Method A			DoE for Method B		
No. Run	Flow Rate	Initial Temp.	No. Run	Flow Rate	Initial Temp.
A-1	1.7	45	B-1	1.8	90
A-2	1.7	40	B-2	1.8	65
A-3	2.0	40	B-3	2.5	65
A-4	2.0	45	B-4	2.5	90
A-5	1.3	35	B-5	1.0	40
A-6	1.3	40	B-6	1.0	65
A-7	1.7	40	B-7	1.8	65
A-8	2.0	35	B-8	2.5	40
A-9	1.7	40	B-9	1.8	65
A-10	1.7	40	B-10	1.8	65
A-11	1.7	35	B-11	1.8	40
A-12	1.3	45	B-12	1.0	90

**Table 5:** Regression ANOVA statistics results in optimization.

		Sum of Squares	DF*	Mean Square	F-Ratio	*p*-Value		Sum of Squares	DF	Mean Square	F-Ratio	*p*-Value
VB	A*	39,784	3	13,261.43	10.4579	<0.01	3I1P	5,270,439.51	4	1,317,609	56.0824	<0.01
2C1P	R*	0.3267	1	0.3267	26.6667	<0.01	1BB	2.1687	2	1.0844	7.0698	0.014
	A	5,078,587	3	1,692,862	10.3662	<0.01		171,839	3	57,279	52.615	<0.01
2CP	R	3.1395	4	0.7849	16.018	<0.01	1B2CE	1.5	1	1.5	5.1546	0.047
	A	658,727	3	219,575.	14.451	<0.01		23,564.55	1	23,564	48.6432	<0.01
BE	R	1.1267	1	1.1267	29.9778	<0.01	11DBE	1.9267	1	1.9267	6.0397	0.034
	A	125,017	3	41,672	13.1523	<0.01		4,647.75	3	1,549	4.2898	0.044
2BP	R	27.9528	3	9.3176	29.3893	<0.01	12DBP	1,631.06	3	543.6883	52.3332	<0.01
	A	<0.01	3	<0.01	41.9238	<0.01		6,255,223.87	4	1,563,805	12.6797	<0.01
3C2M1P	R	5.5787	3	1.8596	14.2526	<0.01	1B3CP	3.1758	2	1.5879	11.8351	<0.01
	A	49,069	3	16,356	32.7338	<0.01		496,361.17	2	248,180	12.5135	<0.01
12DCE	R	38.805	3	12.935	4.3709	0.042	DIM	53.0399	2	26.5199	10.9916	<0.01
	A	21,604	2	10,802	16.9309	<0.01		1,008,620.08	1	1,008,620	8.8133	0.014
2BB	R	7.935	1	7.935	13.79	<0.01	13DBP	0.0158	2	0.0079	52.7139	<0.01
	A	370,632	3	123,544	97.5273	<0.01		150,401.39	2	75,200	4.538	0.048
12DCP	R	17.2294	3	5.7431	10.4585	<0.01	14DBB	2185.18	3	728.3925	12.1323	<0.01
	A	42,511	3	14,170	23.6012	<0.01		172,456.65	2	86,228	8.2038	<0.01
4B1B	R	0.8388	2	0.4194	13.9608	<0.01						
	A	113,294	3	37,764	13.383	<0.01						

A*: peak area, R*: resolution, DF*: degree of freedom.

**Table 6:** Summary of analytical method validation results.

	Specificity	Sensitivity (ppm)		Linearity			Accuracy (%)			Precision (%RSD)	
Name	Resolution	LOD*	LOQ*	R*	Slope	y-Intercept	Low	Mid	High	Repeat- Ability	LOQ Level
Acceptance Criteria	≥1.5	≤0.3 ppm	≤1.0 ppm	≥0.995	-	-	≥85.0 %	≥85.0 %	≥85.0 %	≤10 %RSD	≤10 %RSD
VB	-	0.09	0.29	0.9989	137.91	15.699	90.84	97.90	92.57	4.01	4.35
2C1P	1.5	0.01	0.03	0.9990	1215.68	109.155	87.97	97.64	92.75	5.45	3.35
2CP	5.4	0.03	0.10	0.9993	402.58	29.440	86.79	93.95	90.08	4.51	3.47
BE	4.6	0.01	0.04	0.9997	304.53	37.785	90.72	95.23	89.52	3.69	2.11
2BP	17.4	0.05	0.16	0.9997	170.84	4.359	90.73	96.36	91.21	2.99	1.80
3C2M1P	5.2	0.03	0.09	0.9994	298.47	−1.804	95.29	98.51	93.85	1.94	1.80
12DCE	18.4	0.04	0.13	0.9996	212.38	13.247	99.58	99.16	93.33	3.20	6.43
2BB	12.6	0.01	0.04	0.9996	529.32	8.433	100.64	103.81	98.26	2.07	1.61
12DCP	12.2	0.04	0.14	0.9975	192.29	24.495	98.07	97.78	90.49	3.33	3.31
4B1B	2.8	0.02	0.06	0.9996	347.26	58.163	97.93	97.02	91.62	2.91	1.34
1BB	8.2	0.02	0.05	0.9994	304.80	38.029	98.91	99.47	93.24	2.04	1.93
1B2CE	8.3	0.05	0.16	0.9998	153.73	6.450	100.21	96.61	92.26	4.48	4.31
11DBE	5.9	0.05	0.18	0.9993	158.29	−2.456	101.25	99.50	95.67	3.42	3.81
3I1P	-	0.07	0.25	0.9997	619.84	19.912	96.77	92.01	96.52	3.36	2.28
12DBP	27.8	0.07	0.25	0.9988	321.55	−15.625	94.64	93.67	93.67	6.60	3.97
1B3CP	5.3	0.10	0.33	0.9988	122.00	4.4667	95.70	97.30	95.51	5.41	1.82
DIM	20.6	0.07	0.24	0.9991	318.22	−5.9208	98.81	100.12	98.83	4.54	1.01
13DBP	11.7	0.11	0.38	0.9999	100.11	−0.7792	101.14	102.47	99.88	2.93	1.68
14DBB	63.4	0.07	0.29	0.9993	55.283	3.3083	96.09	97.97	93.34	3.38	2.95

LOD*: Limit of detection, LOQ*: limit of quantitation, R*: correlation coefficient.

## Data Availability

Not applicable.

## Supplementary Materials

The following supporting information can be downloaded at: https://www.mdpi.com/article/10.3390/molecules27144437/s1, Table S1: In silico prediction results for the 144 alkyl halides; Table S2: Screening data of the resolutions; Table S3: Screening data of the peak areas; Table S4: Optimization data of the resolutions; Table S5: Optimization data of the peak areas; Table S6: Applicability of the method on the API Raloxifene hydrochloride (Cas No. 82640-04-8); Table S7: Applicability of the method on the API Febuxostat (Cas No. 144060-53-7); Table S8: Applicability of the method on the Sitagliptin phosphate (Cas No. 654671-78-0); Table S9: Applicability of the method on the Amlodipine camsylate (Cas No. 652969-01-2); Table S10: Applicability of the method on the Ezetimibe (Cas No. 163222-33-1).
