# Supplementary material for: Analytical Method Development for 19 Alkyl Halides as Potential Genotoxic Impurities by Analytical Quality by Design
Source: Molecules. 2022 Jul 11;27(14):4437. doi: 10.3390/molecules27144437 (PMC9320377; doi:10.3390/molecules27144437)
Supplement: Supplementary file 1 [file molecules-27-04437-s001.zip › molecules-1771200-supplementary.pdf]

**Supporting document for the manuscript 'Analytical method development for 19 Alkyl halides as potential genotoxic impurities by analytical Quality by Design' (Manuscript ID : molecules-1771200)**

**Table S1. In silico prediction results for the 144 alkyl halides.**

**Table S2. Screening data of the resolutions.**

**Table S3. Screening data of the peak areas.**

**Table S4. Optimization data of the resolutions.**

**Table S5. Optimization data of the peak areas.**

**Table S6. Applicability of the method on the API Raloxifene hydrochloride (Cas No. 82640-04-8).**

**Table S7. Applicability of the method on the API Febuxostat (Cas No. 144060-53-7).**

**Table S8. Applicability of the method on the API Sitagliptin phosphate (Cas No. 654671-78-0).**

**Table S9. Applicability of the method on the API Amlodipine camsylate (Cas No. 652969-01-2).**

**Table S10. Applicability of the method on the API Ezetimibe (Cas No. 163222-33-1).**

**Table S1. In silico prediction results for 144 alkyl halides.**

| Name | CAS No. | Derek Prediction | Sarah Prediction | VEGA Mutagenicity | ICH M7 Class |
|---|---|---|---|---|---|
| 1-bromopropane | 106-94-5 | PLAUSIBLE | POSITIVE | NEGATIVE | Class 5 |
| 1-bromobutane | 109-65-9 | PLAUSIBLE | POSITIVE | POSITIVE | Class 2 |
| bromoethane | 74-96-4 | PLAUSIBLE | POSITIVE | POSITIVE | Class 1 |
| bromoethene | 593-60-2 | PROBABLE | POSITIVE | POSITIVE | Class 1 |
| bromomethane | 74-83-9 | PLAUSIBLE | POSITIVE | NEGATIVE | Class 5 |
| 2-bromopropane | 75-26-3 | PLAUSIBLE | POSITIVE | POSITIVE | Class 2 |
| 3-bromoprop-1-ene | 106-95-6 | PLAUSIBLE | POSITIVE | POSITIVE | Class 5 |
| (E)-1-bromoprop-1-ene | 590-14-7 | PLAUSIBLE | EQUIVOCAL | POSITIVE | Class 3 |
| 2-bromoprop-1-ene | 557-93-7 | PLAUSIBLE | POSITIVE | POSITIVE | Class 3 |
| 1-bromo-2-methylpropane | 78-77-3 | PLAUSIBLE | NEGATIVE | POSITIVE | Class 5 |
| 2-bromobutane | 78-76-2 | PLAUSIBLE | POSITIVE | POSITIVE | Class 2 |
| 2-bromo-2-methylpropane | 507-19-7 | INACTIVE | POSITIVE | POSITIVE | Class 2 |
| 4-bromobut-1-ene | 5162-44-7 | PLAUSIBLE | POSITIVE | POSITIVE | Class 2 |
| (E)-1-bromobut-2-ene | 4784-77-4 | PLAUSIBLE | POSITIVE | POSITIVE | Class 3 |
| (E)-1-bromobut-1-ene | 31844-98-1 | PLAUSIBLE | NEGATIVE | POSITIVE | Class 3 |
| 3-bromo-2-methylprop-1-ene | 1458-98-6 | PLAUSIBLE | POSITIVE | POSITIVE | Class 2 |

| | | | | | |
|---|---|---|---|---|---|
| 1-bromo-2-methylprop-1-ene | 3017-69-4 | PLAUSIBLE | EQUIVOCAL | POSITIVE | Class 3 |
| 2-bromobut-1-ene | 23074-36-4 | PLAUSIBLE | NEGATIVE | POSITIVE | Class 3 |
| (E)-2-bromobut-2-ene | 3017-71-8 | PLAUSIBLE | EQUIVOCAL | POSITIVE | Class 3 |
| 3-bromobut-1-ene | 22037-73-6 | PLAUSIBLE | POSITIVE | POSITIVE | Class 3 |
| (E)-1-bromobuta-1,3-diene | 89567-73-7 | PLAUSIBLE | POSITIVE | POSITIVE | Class 3 |
| 2-bromobuta-1,3-diene | 1822-86-2 | PLAUSIBLE | POSITIVE | POSITIVE | Class 3 |
| 1-chloropropane | 540-54-5 | PLAUSIBLE | NEGATIVE | POSITIVE | Class 5 |
| 1-chlorobutane | 109-69-3 | PLAUSIBLE | NEGATIVE | NEGATIVE | Class 5 |
| chloroethane | 75-00-3 | PLAUSIBLE | POSITIVE | POSITIVE | Class 1 |
| chloroethene | 75-01-4 | PROBABLE | POSITIVE | POSITIVE | Class 1 |
| chloromethane | 74-87-3 | PLAUSIBLE | POSITIVE | POSITIVE | Class 2 |
| 2-chloropropane | 75-29-6 | PLAUSIBLE | POSITIVE | POSITIVE | Class 2 |
| 3-chloroprop-1-ene | 107-05-1 | PROBABLE | POSITIVE | POSITIVE | Class 5 |
| (E)-1-chloroprop-1-ene | 590-21-6 | PLAUSIBLE | POSITIVE | POSITIVE | Class 1 |
| 2-chloroprop-1-ene | 557-98-2 | PLAUSIBLE | POSITIVE | POSITIVE | Class 2 |
| 1-chloro-2-methylpropane | 513-36-0 | PLAUSIBLE | POSITIVE | POSITIVE | Class 3 |
| 2-chlorobutane | 78-86-4 | PLAUSIBLE | NEGATIVE | POSITIVE | Class 5 |
| 2-chloro-2-methylpropane | 507-20-0 | INACTIVE | NEGATIVE | POSITIVE | Class 5 |
| 4-chlorobut-1-ene | 927-73-1 | PLAUSIBLE | NEGATIVE | POSITIVE | Class 5 |

| | | | | | |
|---|---|---|---|---|---|
| (E)-1-chlorobut-2-ene | 4894-61-5 | PLAUSIBLE | POSITIVE | POSITIVE | Class 2 |
| (E)-1-chlorobut-1-ene | 4461-42-1 | PLAUSIBLE | POSITIVE | POSITIVE | Class 2 |
| 3-chloro-2-methylprop-1-ene | 563-47-3 | PLAUSIBLE | POSITIVE | POSITIVE | Class 1 |
| 1-chloro-2-methylprop-1-ene | 513-37-1 | PLAUSIBLE | POSITIVE | POSITIVE | Class 3 |
| 2-chlorobut-1-ene | 52844-20-9 | PLAUSIBLE | POSITIVE | POSITIVE | Class 3 |
| (E)-2-chlorobut-2-ene | 4461-41-0 | PLAUSIBLE | POSITIVE | POSITIVE | Class 2 |
| 3-chlorobut-1-ene | 563-52-0 | PLAUSIBLE | POSITIVE | POSITIVE | Class 2 |
| (E)-1-chlorobuta-1,3-diene | 627-22-5 | PLAUSIBLE | POSITIVE | POSITIVE | Class 2 |
| 2-chlorobuta-1,3-diene | 126-99-8 | PLAUSIBLE | POSITIVE | NEGATIVE | Class 1 |
| 1-iodopropane | 107-08-4 | PLAUSIBLE | POSITIVE | POSITIVE | Class 3 |
| 1-iodobutane | 542-69-8 | PLAUSIBLE | POSITIVE | POSITIVE | Class 3 |
| iodoethane | 75-03-6 | PROBABLE | POSITIVE | POSITIVE | Class 2 |
| iodoethene | 593-66-8 | PLAUSIBLE | NEGATIVE | POSITIVE | Class 3 |
| iodomethane | 74-88-4 | PLAUSIBLE | POSITIVE | POSITIVE | Class 2 |
| 2-iodopropane | 75-30-9 | PLAUSIBLE | POSITIVE | POSITIVE | Class 3 |
| 3-iodoprop-1-ene | 556-56-9 | PLAUSIBLE | POSITIVE | POSITIVE | Class 2 |
| (E)-1-iodoprop-1-ene | 7796-54-5 | PLAUSIBLE | EQUIVOCAL | POSITIVE | Class 3 |
| 2-iodoprop-1-ene | 4375-96-6 | PLAUSIBLE | EQUIVOCAL | POSITIVE | Class 3 |
| 1-iodo-2-methylpropane | 513-38-2 | PLAUSIBLE | POSITIVE | POSITIVE | Class 3 |

| | | | | | |
|---|---|---|---|---|---|
| 2-iodobutane | 513-48-4 | PLAUSIBLE | POSITIVE | POSITIVE | Class 2 |
| 2-iodo-2-methylpropane | 558-17-8 | INACTIVE | POSITIVE | POSITIVE | Class 2 |
| 4-iodobut-1-ene | 7766-51-0 | PLAUSIBLE | POSITIVE | POSITIVE | Class 3 |
| (E)-1-iodobut-2-ene | 627-24-7 | PLAUSIBLE | EQUIVOCAL | POSITIVE | Class 3 |
| (E)-1-iodobut-1-ene | 104164-79-6 | PLAUSIBLE | NEGATIVE | POSITIVE | Class 3 |
| 3-iodo-2-methylprop-1-ene | 3756-30-7 | PLAUSIBLE | EQUIVOCAL | POSITIVE | Class 3 |
| 1-iodo-2-methylprop-1-ene | 20687-01-8 | PLAUSIBLE | EQUIVOCAL | POSITIVE | Class 3 |
| 2-iodobut-1-ene | 24308-61-0 | PLAUSIBLE | NEGATIVE | POSITIVE | Class 3 |
| (E)-2-iodobut-2-ene | 62154-76-1 | PLAUSIBLE | EQUIVOCAL | POSITIVE | Class 3 |
| 3-iodobut-1-ene | 62154-74-9 | PLAUSIBLE | POSITIVE | POSITIVE | Class 3 |
| (E)-1-iodobuta-1,3-diene | 6088-91-1 | PLAUSIBLE | POSITIVE | POSITIVE | Class 3 |
| 2-iodobuta-1,3-diene | 19221-28-4 | PLAUSIBLE | POSITIVE | POSITIVE | Class 3 |
| 1-bromo-2-chloroethane | 107-04-0 | PLAUSIBLE | POSITIVE | POSITIVE | Class 2 |
| 1-bromo-3-chloropropane | 109-70-6 | PLAUSIBLE | POSITIVE | POSITIVE | Class 2 |
| bromochloromethane | 74-97-5 | PLAUSIBLE | POSITIVE | POSITIVE | Class 2 |
| 1-bromo-1-chloroethane | 593-96-4 | PLAUSIBLE | POSITIVE | POSITIVE | Class 3 |
| 2-bromo-1-chloropropane | 3017-95-6 | PLAUSIBLE | POSITIVE | POSITIVE | Class 3 |
| 2-bromo-2-chloropropane | 2310-98-7 | INACTIVE | POSITIVE | POSITIVE | Class 3 |
| 1-bromo-4-chlorobutane | 6940-78-9 | PLAUSIBLE | POSITIVE | POSITIVE | Class 3 |

| | | | | | |
|---|---|---|---|---|---|
| 1-chloro-2-iodoethane | 624-70-4 | PLAUSIBLE | POSITIVE | POSITIVE | Class 3 |
| 1-chloro-3-iodopropane | 6940-76-7 | PLAUSIBLE | POSITIVE | POSITIVE | Class 3 |
| chloroiodomethane | 593-71-5 | PLAUSIBLE | POSITIVE | POSITIVE | Class 3 |
| 1-chloro-1-iodoethane | 594-00-3 | PLAUSIBLE | POSITIVE | POSITIVE | Class 3 |
| 1-chloro-2-iodopropane | 12596099 | PLAUSIBLE | POSITIVE | POSITIVE | Class 3 |
| 2-chloro-2-iodopropane | 116632-32-7 | INACTIVE | POSITIVE | POSITIVE | Class 3 |
| 1-chloro-4-iodobutane | 10297-05-9 | PLAUSIBLE | POSITIVE | POSITIVE | Class 3 |
| 1-bromo-2-iodoethane | 590-16-9 | PLAUSIBLE | POSITIVE | POSITIVE | Class 3 |
| 1-bromo-3-iodopropane | 22306-36-1 | PLAUSIBLE | POSITIVE | POSITIVE | Class 3 |
| bromoiodomethane | 557-68-6 | PLAUSIBLE | POSITIVE | POSITIVE | Class 3 |
| 1-bromo-1-iodoethane | 21931755 | PLAUSIBLE | POSITIVE | POSITIVE | Class 3 |
| 1-bromo-2-iodopropane | 74236-17-2 | PLAUSIBLE | POSITIVE | POSITIVE | Class 3 |
| 2-bromo-2-iodopropane | 23080714 | INACTIVE | POSITIVE | POSITIVE | Class 3 |
| 1-bromo-4-iodobutane | 89044-65-5 | PLAUSIBLE | POSITIVE | POSITIVE | Class 3 |
| 1,2-dichloroethane | 107-06-2 | PLAUSIBLE | POSITIVE | POSITIVE | Class 1 |
| 1,3-dichloropropane | 142-28-9 | PLAUSIBLE | POSITIVE | POSITIVE | Class 3 |
| dichloromethane | 75-09-2 | PLAUSIBLE | POSITIVE | POSITIVE | Class 1 |
| 1,1-dichloroethane | 75-34-3 | INACTIVE | NEGATIVE | NEGATIVE | Class 5 |
| 1,2-dichloropropane | 78-87-5 | PLAUSIBLE | POSITIVE | POSITIVE | Class 1 |
| 2,2-dichloropropane | 594-20-7 | INACTIVE | NEGATIVE | NEGATIVE | Class 5 |

| | | | | | |
|---|---|---|---|---|---|
| 1,4-dichlorobutane | 110-56-5 | PLAUSIBLE | POSITIVE | POSITIVE | Class 3 |
| 1,2-dibromoethane | 106-93-4 | PLAUSIBLE | POSITIVE | POSITIVE | Class 1 |
| 1,3-dibromopropane | 109-64-8 | PLAUSIBLE | POSITIVE | NEGATIVE | Class 2 |
| dibromomethane | 74-95-3 | PROBABLE | POSITIVE | POSITIVE | Class 2 |
| 1,1-dibromoethane | 557-91-5 | PLAUSIBLE | POSITIVE | NEGATIVE | Class 2 |
| 1,2-dibromopropane | 78-75-1 | PLAUSIBLE | POSITIVE | POSITIVE | Class 2 |
| 2,2-dibromopropane | 594-16-1 | INACTIVE | POSITIVE | POSITIVE | Class 3 |
| 1,4-dibromobutane | 110-52-1 | PLAUSIBLE | POSITIVE | POSITIVE | Class 2 |
| 1,2-diiodoethane | 624-73-7 | PLAUSIBLE | NEGATIVE | POSITIVE | Class 5 |
| 1,3-diiodopropane | 627-31-6 | PLAUSIBLE | POSITIVE | POSITIVE | Class 3 |
| diiodomethane | 75-11-6 | PROBABLE | POSITIVE | NEGATIVE | Class 2 |
| 1,1-diiodoethane | 594-02-5 | PLAUSIBLE | POSITIVE | POSITIVE | Class 3 |
| 1,2-diiodopropane | 598-29-8 | PLAUSIBLE | POSITIVE | POSITIVE | Class 3 |
| 2,2-diiodopropane | 630-13-7 | INACTIVE | POSITIVE | NEGATIVE | Class 3 |
| 1,4-diiodobutane | 628-21-7 | PLAUSIBLE | POSITIVE | POSITIVE | Class 3 |
| 2-chloroacetyl chloride | 286367-76-8 | PLAUSIBLE | NEGATIVE | POSITIVE | Class 5 |
| 3-chloropropanoyl chloride | 625-36-5 | PLAUSIBLE | POSITIVE | POSITIVE | Class 2 |
| 4-chlorobutanoyl chloride | 4635-59-0 | PLAUSIBLE | EQUIVOCAL | POSITIVE | Class 3 |
| 1,3-dichloropropan-2-one | 534-07-6 | PLAUSIBLE | POSITIVE | POSITIVE | Class 2 |

| | | | | | |
|---|---|---|---|---|---|
| propionyl chloride | 79-03-8 | EQUIVOCAL | NEGATIVE | POSITIVE | Class 5 |
| butyryl chloride | 141-75-3 | EQUIVOCAL | NEGATIVE | POSITIVE | Class 3 |
| pentanoyl chloride | 638-29-9 | EQUIVOCAL | NEGATIVE | POSITIVE | Class 5 |
| 1-chlorobutan-2-one | 616-27-3 | INACTIVE | EQUIVOCAL | POSITIVE | Inconclusive |
| 1-chloropropan-2-one | 78-95-5 | INACTIVE | POSITIVE | NEGATIVE | Class 3 |
| 4-chlorobutan-2-one | 6322-49-2 | PLAUSIBLE | NEGATIVE | POSITIVE | Class 3 |
| 5-chloropentan-2-one | 5891-21-4 | PLAUSIBLE | NEGATIVE | POSITIVE | Class 3 |
| 1-chlorobutan-2-one | 616-27-3 | INACTIVE | EQUIVOCAL | POSITIVE | Inconclusive |
| 2-bromoacetyl bromide | 598-21-0 | PLAUSIBLE | POSITIVE | POSITIVE | Class 2 |
| 3-bromopropanoyl bromide | 7623-16-7 | PLAUSIBLE | POSITIVE | POSITIVE | Class 3 |
| 4-bromobutanoyl bromide | 56489-06-6 | PLAUSIBLE | POSITIVE | POSITIVE | Class 3 |
| 1,3-dibromopropan-2-one | 816-39-7 | PLAUSIBLE | POSITIVE | POSITIVE | Class 3 |
| propionyl bromide | 598-22-1 | EQUIVOCAL | NEGATIVE | POSITIVE | Class 3 |
| butyryl bromide | 5856-82-6 | EQUIVOCAL | NEGATIVE | POSITIVE | Class 3 |
| pentanoyl bromide | 1889-26-5 | EQUIVOCAL | NEGATIVE | POSITIVE | Class 3 |
| 1-bromobutan-2-one | 816-40-0 | INACTIVE | POSITIVE | POSITIVE | Class 3 |
| 1-bromopropan-2-one | 598-31-2 | INACTIVE | POSITIVE | POSITIVE | Class 3 |
| 4-bromobutan-2-one | 28509-46-8 | PLAUSIBLE | EQUIVOCAL | POSITIVE | Class 3 |
| 5-bromopentan-2-one | 3884-71-7 | PLAUSIBLE | EQUIVOCAL | POSITIVE | Class 3 |

| | | | | | |
|---|---|---|---|---|---|
| 1-bromobutan-2-one | 816-40-0 | INACTIVE | POSITIVE | POSITIVE | Class 3 |
| 2-iodoacetyl iodide | 18506236 | PLAUSIBLE | OUTSIDE DOMAIN | POSITIVE | Class 3 |
| 3-iodopropanoyl iodide | 12715997 | PLAUSIBLE | OUTSIDE DOMAIN | POSITIVE | Class 3 |
| 4-iodobutanoyl iodide | 14641657 | PLAUSIBLE | OUTSIDE DOMAIN | POSITIVE | Class 3 |
| 1,3-diiodopropan-2-one | 6305-40-4 | PLAUSIBLE | POSITIVE - 1% | POSITIVE | Class 3 |
| propionyl iodide | 12652484 | EQUIVOCAL | OUTSIDE DOMAIN | POSITIVE | Class 3 |
| butyryl iodide | 10899590 | EQUIVOCAL | OUTSIDE DOMAIN | POSITIVE | Class 3 |
| pentanoyl iodide | 12550986 | EQUIVOCAL | OUTSIDE DOMAIN | POSITIVE | Class 3 |
| 1-iodobutan-2-one | 78389-71-6 | INACTIVE | EQUIVOCAL | NEGATIVE | Inconclusive |
| 1-iodopropan-2-one | 3019-04-3 | INACTIVE | POSITIVE | NEGATIVE | Class 3 |
| 4-iodobutan-2-one | 57688-61-6 | PLAUSIBLE | NEGATIVE | NEGATIVE | Class 3 |
| 5-iodopentan-2-one | 3695-29-2 | PLAUSIBLE | NEGATIVE | NEGATIVE | Class 3 |
| 1-iodobutan-2-one | 78389-71-6 | INACTIVE | EQUIVOCAL | NEGATIVE | Inconclusive |

**Table S2. Screening data of the resolutions.**

| 2C1P | 2CP | BE | 2BP | 3C2 M1P | 12 DCE | 2BB | 12 DCP | 4B1B | 1BB | 1B 2CE | 11 DBE | 12 DBP | 1B 3CP | DIM | 13 DBP | 14 DBB |
|---|---|---|---|---|---|---|---|---|---|---|---|---|---|---|---|---|
| 1.6 | 5.3 | 4.8 | 18.6 | 6.1 | 17.4 | 11.4 | 12.0 | 2.8 | 8.1 | 8.3 | 6.0 | 58.4 | 6.8 | 19.8 | 14.9 | 45.1 |
| 0.5 | 2.5 | 2.4 | 8.0 | 1.6 | 10.0 | 7.2 | 6.0 | 1.2 | 4.7 | 5.8 | 4.2 | 29.9 | 6.4 | 23.2 | 12.8 | 64.8 |
| 1.0 | 4.0 | 3.7 | 13.4 | 3.4 | 13.7 | 8.8 | 8.5 | 1.7 | 5.7 | 5.7 | 3.6 | 72.2 | 7.3 | 15.8 | 8.7 | 30.7 |
| 1.6 | 5.7 | 5.0 | 20.0 | 5.4 | 18.4 | 12.3 | 11.7 | 2.9 | 8.4 | 8.5 | 6.1 | 58.4 | 6.5 | 19.0 | 14.7 | 52.7 |
| 1.5 | 6.0 | 5.0 | 19.3 | 6.5 | 20.4 | 13.7 | 14.3 | 3.6 | 9.6 | 9.3 | 6.7 | 56.4 | 6.6 | 18.8 | 15.1 | 41.2 |
| 1.6 | 5.3 | 4.6 | 19.0 | 6.9 | 21.9 | 13.9 | 14.5 | 3.3 | 8.8 | 9.0 | 6.7 | 60.2 | 6.8 | 16.1 | 13.1 | 44.0 |
| 1.3 | 4.1 | 3.8 | 13.4 | 3.4 | 14.5 | 9.9 | 8.8 | 1.8 | 6.1 | 6.2 | 4.1 | 69.0 | 6.9 | 18.0 | 7.7 | 28.7 |
| 0.8 | 2.5 | 2.5 | 8.6 | 1.9 | 10.7 | 8.0 | 7.9 | 1.7 | 5.9 | 6.5 | 4.8 | 31.0 | 6.0 | 22.4 | 12.5 | 49.3 |
| 1.1 | 3.5 | 3.3 | 11.8 | 3.5 | 14.7 | 10.9 | 10.7 | 2.4 | 8.1 | 8.7 | 6.3 | 38.5 | 5.8 | 19.3 | 13.1 | 58.9 |
| 1.1 | 3.5 | 3.2 | 11.5 | 3.3 | 14.5 | 10.9 | 10.4 | 2.3 | 7.6 | 8.2 | 5.9 | 39.4 | 6.3 | 20.4 | 13.9 | 53.7 |
| 1.1 | 3.4 | 3.0 | 11.8 | 3.2 | 14.6 | 11.0 | 10.7 | 2.4 | 8.0 | 8.3 | 6.0 | 39.7 | 5.9 | 18.2 | 12.1 | 54.3 |
| 1.0 | 2.2 | 1.5 | 5.7 | 1.0 | 7.5 | 5.6 | 5.1 | 0.8 | 3.7 | 4.2 | 2.9 | 26.3 | 4.1 | 17.9 | 8.0 | 27.9 |
| 0.5 | 2.4 | 2.2 | 7.4 | 1.9 | 9.8 | 7.5 | 6.3 | 1.2 | 4.6 | 5.4 | 3.8 | 32.5 | 6.7 | 24.9 | 14.1 | 63.8 |
| 1.1 | 2.0 | 1.6 | 5.7 | 1.0 | 6.6 | 5.3 | 5.4 | 1.1 | 3.5 | 4.4 | 3.1 | 28.9 | 4.2 | 17.9 | 9.4 | 34.7 |
| 0.8 | 2.0 | 2.0 | 5.4 | 1.1 | 7.0 | 5.4 | 5.2 | 1.1 | 4.0 | 4.3 | 3.1 | 28.7 | 4.7 | 18.8 | 10.5 | 38.3 |
| 0.8 | 2.0 | 1.6 | 5.7 | 1.0 | 6.1 | 4.9 | 5.1 | 0.9 | 3.1 | 4.1 | 2.6 | 31.0 | 4.7 | 18.5 | 9.9 | 36.9 |
| 0.5 | 2.6 | 2.1 | 7.3 | 1.6 | 10.1 | 7.8 | 6.7 | 1.3 | 5.0 | 6.4 | 4.8 | 29.7 | 6.4 | 24.9 | 14.3 | 64.6 |
| 1.0 | 3.4 | 3.1 | 11.3 | 3.1 | 13.8 | 10.1 | 9.4 | 1.9 | 6.1 | 7.1 | 5.3 | 39.4 | 5.8 | 17.8 | 11.7 | 54.8 |
| 0.9 | 4.2 | 3.8 | 13.8 | 3.6 | 13.4 | 9.0 | 8.7 | 1.7 | 5.7 | 5.6 | 3.9 | 56.9 | 5.0 | 13.6 | 8.1 | 32.6 |
| 1.2 | 3.9 | 3.8 | 14.2 | 3.4 | 11.2 | 7.1 | 8.0 | 1.7 | 5.0 | 4.9 | 3.4 | 67.1 | 6.1 | 13.0 | 7.6 | 31.0 |

**Table S3. Screening data of peak areas.**

| VB | 2C1P | 2CP | BE | 2BP | 3C2 M1P | 12 DCE | 2BB | 12 DCP | 4B1B | 1BB | 1B 2CE | 11 DBE | 3I1P | 12 DBP | 1B 3CP | DIM | 13 DBP | 14 DBB |
|---|---|---|---|---|---|---|---|---|---|---|---|---|---|---|---|---|---|---|
| 507 | 4586 | 1411 | 2195 | 476 | 595 | 890 | 1306 | 576 | 802 | 1000 | 451 | 372 | 5564 | 2761 | 978 | 2294 | 714 | 319 |
| 236 | 4589 | 1329 | 2426 | 564 | 736 | 961 | 1645 | 602 | 1003 | 1242 | 495 | 400 | 4962 | 2473 | 890 | 2296 | 699 | 316 |
| 185 | 1708 | 652 | 1002 | 298 | 374 | 585 | 896 | 291 | 608 | 679 | 214 | 204 | 6422 | 2768 | 1175 | 1681 | 653 | 300 |
| 501 | 4574 | 1356 | 2395 | 495 | 653 | 852 | 1381 | 515 | 853 | 1102 | 432 | 370 | 5518 | 2785 | 1034 | 2549 | 726 | 344 |
| 440 | 3858 | 1145 | 2132 | 456 | 623 | 881 | 1287 | 525 | 864 | 1051 | 452 | 376 | 5410 | 2759 | 972 | 2339 | 730 | 344 |
| 506 | 4604 | 1362 | 2367 | 506 | 658 | 847 | 1352 | 520 | 853 | 1102 | 422 | 369 | 5657 | 2815 | 994 | 2433 | 763 | 366 |
| 263 | 2238 | 850 | 1166 | 371 | 410 | 550 | 979 | 324 | 589 | 723 | 289 | 234 | 6775 | 3132 | 1304 | 1952 | 758 | 313 |
| 217 | 4692 | 1364 | 2317 | 552 | 724 | 955 | 1536 | 608 | 958 | 1170 | 508 | 421 | 5129 | 2704 | 989 | 2524 | 791 | 380 |
| 518 | 7075 | 2309 | 2982 | 722 | 974 | 1163 | 2263 | 902 | 1342 | 1808 | 721 | 508 | 6906 | 3456 | 1393 | 2905 | 998 | 474 |
| 509 | 6714 | 2175 | 2853 | 713 | 962 | 1171 | 2199 | 880 | 1351 | 1754 | 759 | 493 | 7071 | 3549 | 1400 | 2917 | 978 | 454 |
| 568 | 7846 | 2546 | 3136 | 770 | 1021 | 1126 | 2356 | 855 | 1370 | 1859 | 775 | 522 | 6881 | 3549 | 1443 | 3037 | 1035 | 475 |
| 63 | 1986 | 710 | 1469 | 317 | 394 | 610 | 932 | 364 | 608 | 696 | 300 | 229 | 6232 | 2659 | 1155 | 1862 | 694 | 307 |
| 243 | 4867 | 1418 | 2333 | 566 | 682 | 946 | 1511 | 556 | 863 | 1063 | 490 | 393 | 4725 | 2449 | 900 | 2373 | 717 | 332 |
| 29 | 1464 | 613 | 1284 | 271 | 362 | 719 | 897 | 255 | 606 | 640 | 236 | 222 | 5847 | 2538 | 1156 | 1907 | 746 | 357 |
| 135 | 2241 | 856 | 1126 | 362 | 410 | 668 | 973 | 322 | 543 | 736 | 327 | 228 | 6092 | 2587 | 1117 | 1810 | 655 | 295 |
| 113 | 2356 | 859 | 1181 | 363 | 435 | 766 | 1046 | 320 | 735 | 710 | 394 | 233 | 5306 | 2498 | 1083 | 1801 | 681 | 311 |
| 233 | 4096 | 1228 | 2087 | 511 | 600 | 938 | 1314 | 536 | 806 | 925 | 457 | 385 | 5326 | 2646 | 978 | 2621 | 807 | 395 |
| 497 | 6016 | 2045 | 2605 | 672 | 864 | 1126 | 2040 | 784 | 1159 | 1553 | 678 | 469 | 6949 | 3503 | 1404 | 2884 | 976 | 438 |
| 247 | 1980 | 774 | 1008 | 316 | 396 | 603 | 909 | 326 | 544 | 708 | 331 | 238 | 6964 | 3537 | 1540 | 2440 | 1011 | 490 |
| 160 | 1279 | 548 | 789 | 248 | 331 | 698 | 770 | 305 | 504 | 569 | 256 | 219 | 7434 | 3406 | 1445 | 2148 | 854 | 397 |

**Table S4. Optimization data of the resolutions.**

| 2C1P | 2CP | BE | 2BP | 3C2 M1P | 12 DCE | 2BB | 12 DCP | 4B1B | 1BB | 1B 2CE | 11 DBE | 12 DBP | 1B 3CP | DIM | 13 DBP | 14 DBB |
|---|---|---|---|---|---|---|---|---|---|---|---|---|---|---|---|---|
| 1.1 | 4.1 | 3.6 | 13.3 | 4.1 | 17.8 | 11.6 | 10.2 | 2.5 | 7.8 | 8.0 | 5.8 | 26.1 | 5.1 | 20.9 | 12 | 60.2 |
| 1.3 | 4.3 | 3.8 | 15.7 | 4.1 | 15.7 | 11.2 | 10.8 | 2.5 | 7.6 | 6.8 | 4.6 | 40 | 5.2 | 17.4 | 11.6 | 57.2 |
| 1.4 | 4.2 | 3.9 | 15.5 | 4.7 | 17.8 | 12.1 | 11.5 | 2.9 | 8.3 | 8.1 | 5.9 | 38 | 6.7 | 22.1 | 15.4 | 53.3 |
| 0.9 | 3.7 | 3.7 | 14.3 | 3.4 | 13.5 | 10.0 | 9.9 | 2.4 | 7.2 | 7.4 | 4.8 | 28.4 | 5.4 | 19.6 | 11.5 | 61.3 |
| 1.4 | 4.5 | 4.1 | 15.4 | 3.9 | 14.5 | 12.0 | 11.4 | 2.7 | 8.0 | 8.0 | 5.8 | 64.1 | 4.8 | 13.4 | 7.9 | 29.5 |
| 1.3 | 4.0 | 4.0 | 13.1 | 4.3 | 16.9 | 11.2 | 10.8 | 2.6 | 7.8 | 8.0 | 5.9 | 50.4 | 5.1 | 14.2 | 7.6 | 30.3 |
| 1.2 | 4.3 | 3.9 | 14.8 | 4.0 | 15.0 | 11.0 | 10.7 | 2.5 | 7.7 | 7.9 | 5.6 | 40.6 | 5.5 | 18.4 | 12.1 | 58.9 |
| 1.6 | 5.6 | 4.6 | 17.7 | 6.0 | 21.1 | 13.0 | 13.7 | 3.2 | 8.5 | 8.7 | 6.3 | 49.8 | 43.3 | 53.5 | 21.5 | 13.7 |
| 1.4 | 4.5 | 3.9 | 14.5 | 4.7 | 17.7 | 12.5 | 12.9 | 3.0 | 8.5 | 8.8 | 6.6 | 37.4 | 4.9 | 15.6 | 10.5 | 52.6 |
| 1.1 | 4.0 | 3.6 | 13.9 | 4.2 | 15.2 | 11.2 | 11.6 | 2.6 | 7.3 | 7.8 | 5.6 | 42 | 5.5 | 18.4 | 12.4 | 60.1 |
| 1.4 | 5.1 | 4.6 | 17.7 | 5.6 | 20.5 | 14.1 | 13.9 | 3.2 | 8.6 | 8.8 | 6.6 | 63 | 6 | 16.1 | 11.9 | 48.6 |
| 1.0 | 3.6 | 3.4 | 12.4 | 3.5 | 14.7 | 10.6 | 9.7 | 2.2 | 6.8 | 7.1 | 4.7 | 28.8 | 4.2 | 18.3 | 9.9 | 36.9 |

**Table S5. Optimization data of the peak areas.**

| VB | 2C1P | 2CP | BE | 2BP | 3C2 M1P | 12 DCE | 2BB | 12 DCP | 4B1B | 1BB | 1B 2CE | 11 DBE | 3I1P | 12 DBP | 1B 3CP | DIM | 13 DBP | 14 DBB |
|---|---|---|---|---|---|---|---|---|---|---|---|---|---|---|---|---|---|---|
| 566 | 5958 | 1780 | 1207 | 492 | 715 | 878 | 1464 | 604 | 899 | 967 | 491 | 405 | 5784 | 3171 | 1255 | 3199 | 961 | 505 |
| 580 | 5597 | 1826 | 1247 | 527 | 780 | 958 | 1572 | 615 | 921 | 1047 | 505 | 413 | 6364 | 3119 | 1138 | 2913 | 864 | 413 |
| 483 | 4512 | 1341 | 1024 | 424 | 642 | 831 | 1226 | 549 | 826 | 842 | 443 | 397 | 5319 | 2774 | 848 | 2846 | 725 | 385 |
| 487 | 4057 | 1223 | 953 | 407 | 638 | 850 | 1208 | 529 | 802 | 827 | 456 | 400 | 4700 | 2385 | 796 | 2563 | 663 | 295 |
| 615 | 5237 | 1659 | 1109 | 479 | 780 | 920 | 1637 | 705 | 1066 | 1142 | 577 | 445 | 6856 | 3742 | 1538 | 2662 | 991 | 434 |
| 623 | 5525 | 1754 | 1155 | 512 | 821 | 950 | 1717 | 727 | 1155 | 1203 | 600 | 461 | 7235 | 3549 | 1548 | 2669 | 1011 | 484 |
| 641 | 6232 | 1962 | 1277 | 535 | 786 | 910 | 1540 | 638 | 969 | 1070 | 512 | 430 | 6344 | 3759 | 1485 | 3806 | 1229 | 577 |
| 472 | 4357 | 1283 | 977 | 401 | 629 | 821 | 1180 | 527 | 840 | 823 | 433 | 383 | 1488 | 879 | 5293 | 3095 | 3386 | 885 |
| 583 | 5764 | 1806 | 1208 | 579 | 762 | 936 | 1509 | 643 | 1003 | 1032 | 529 | 438 | 6665 | 3804 | 1455 | 3673 | 1138 | 493 |
| 662 | 6321 | 2001 | 1301 | 557 | 823 | 935 | 1622 | 671 | 1046 | 1108 | 536 | 453 | 6723 | 3253 | 1177 | 2939 | 861 | 361 |
| 522 | 4959 | 1518 | 1088 | 469 | 759 | 960 | 1462 | 671 | 1071 | 1056 | 530 | 455 | 5787 | 3166 | 1235 | 3035 | 1009 | 643 |
| 641 | 6070 | 1876 | 1223 | 514 | 763 | 939 | 1675 | 652 | 1016 | 1116 | 537 | 409 | 6086 | 2931 | 1264 | 2251 | 827 | 419 |

**Table S6. Applicability of the method on the API Raloxifene hydrochloride (Cas No. 82640-04-8).**

| | Present amount in API (ppm, P) | Theoretical Spiked amount (ppm, T) | Actual detection Amount (ppm, A) | Recovery ((A-P) / T * 100) (%) | Precision (%RSD) | Specificity (Resolution) | LOD (ppm) | LOQ (ppm) |
|---|---|---|---|---|---|---|---|---|
| VB | 0 | 2.08 | 1.86 | 89.4 | 5.59 | - | 0.039 | 0.129 |
| 2C1P | 0 | 2.00 | 1.72 | 86.0 | 7.06 | 1.2 | 0.004 | 0.014 |
| 2CP | 0 | 2.10 | 1.83 | 87.1 | 6.28 | 4.2 | 0.012 | 0.041 |
| BE | 0 | 2.05 | 1.77 | 86.3 | 5.02 | 3.8 | 0.01 | 0.034 |
| 2BP | 0 | 2.20 | 2.05 | 93.2 | 4.19 | 14.5 | 0.023 | 0.078 |
| 3C2M1P | 0 | 2.21 | 2.12 | 95.9 | 2.63 | 5.1 | 0.012 | 0.042 |
| 12DCE | 0 | 2.17 | 2.06 | 94.9 | 0.21 | 18 | 0.019 | 0.063 |
| 2BB | 0 | 2.09 | 2.00 | 95.7 | 2.63 | 12.5 | 0.006 | 0.021 |
| 12DCP | 0 | 2.13 | 1.96 | 92.0 | 2.45 | 12.1 | 0.018 | 0.061 |
| 4B1B | 0 | 2.20 | 2.06 | 93.6 | 1.61 | 2.9 | 0.009 | 0.029 |
| 1BB | 0 | 2.10 | 1.93 | 91.9 | 2.69 | 8.6 | 0.009 | 0.03 |
| 1B2CE | 0 | 2.14 | 1.95 | 91.1 | 2.39 | 8.6 | 0.023 | 0.077 |
| 11DBE | 0 | 2.17 | 2.07 | 95.4 | 1.38 | 6.1 | 0.023 | 0.077 |
| 3I1P | 0 | 4.95 | 4.48 | 90.5 | 10.66 | - | 0.008 | 0.028 |
| 12DBP | 0 | 5.42 | 5.46 | 100.7 | 4.49 | 18.5 | 0.006 | 0.019 |
| 1B3CP | 0 | 5.22 | 5.26 | 100.8 | 1.81 | 5.3 | 0.014 | 0.046 |
| DIM | 0 | 5.17 | 5.31 | 102.7 | 3.93 | 20.3 | 0.005 | 0.017 |
| 13DBP | 0 | 5.00 | 5.08 | 101.6 | 4.7 | 11.6 | 0.015 | 0.050 |
| 14DBB | 0 | 5.02 | 4.89 | 97.4 | 4.32 | 63.8 | 0.017 | 0.057 |

**Table S7. Applicability of the method on the API Febuxostat (Cas No. 144060-53-7).**

| | Present amount in API (ppm, P) | Theoretical Spiked amount (ppm, T) | Actual detection Amount (ppm, A) | Recovery ((A-P) / T * 100) (%) | Precision (%RSD) | Specificity (Resolution) | LOD (ppm) | LOQ (ppm) |
|---|---|---|---|---|---|---|---|---|
| VB | 0 | 2.08 | 1.99 | 95.7 | 1.64 | - | 0.049 | 0.163 |
| 2C1P | 0 | 2.18 | 1.98 | 90.8 | 1.18 | 1.1 | 0.005 | 0.016 |
| 2CP | 0 | 2.30 | 2.15 | 93.5 | 1.37 | 4.2 | 0.014 | 0.048 |
| BE | 0 | 2.16 | 2.38 | 110.2 | 0.57 | 3.8 | 0.008 | 0.027 |
| 2BP | 0 | 2.13 | 2.00 | 93.9 | 0.93 | 14.7 | 0.029 | 0.097 |
| 3C2M1P | 0 | 2.19 | 1.90 | 86.8 | 0.56 | 16.7 | 0.021 | 0.068 |
| 12DCE | 0 | 2.13 | 2.26 | 106.1 | 0.7 | 18.1 | 0.018 | 0.061 |
| 2BB | 0 | 2.17 | 1.69 | 77.9 | 0.26 | 12.7 | 0.006 | 0.021 |
| 12DCP | 0 | 2.12 | 1.88 | 88.7 | 2.38 | 9.4 | 0.027 | 0.089 |
| 4B1B | 0 | 2.09 | 1.77 | 84.7 | 1.51 | 2.8 | 0.011 | 0.037 |
| 1BB | 0 | 2.26 | 2.08 | 92.0 | 0.71 | 8.3 | 0.009 | 0.032 |
| 1B2CE | 0 | 2.19 | 2.15 | 98.2 | 1.85 | 8.4 | 0.034 | 0.127 |
| 11DBE | 0 | 2.13 | 2.06 | 96.7 | 2.09 | 6.1 | 0.033 | 0.127 |
| 3I1P | 0 | 5.24 | 5.22 | 99.6 | 2.24 | - | 0.008 | 0.026 |
| 12DBP | 0 | 5.32 | 5.22 | 98.1 | 2.47 | 26.4 | 0.006 | 0.020 |
| 1B3CP | 0 | 5.39 | 4.88 | 90.5 | 0.81 | 6.0 | 0.013 | 0.045 |
| DIM | 0 | 5.25 | 5.21 | 99.2 | 1.60 | 14.3 | 0.005 | 0.015 |
| 13DBP | 0 | 5.65 | 5.41 | 95.8 | 1.66 | 13.0 | 0.016 | 0.053 |
| 14DBB | 0 | 5.16 | 5.09 | 98.6 | 0.20 | 69.4 | 0.023 | 0.076 |

**Table S8. Applicability of the method on the API Sitagliptin phosphate (Cas No. 654671-78-0).**

| | Present amount in API (ppm, P) | Theoretical Spiked amount (ppm, T) | Actual detection Amount (ppm, A) | Recovery ((A-P) / T * 100) (%) | Precision (%RSD) | Specificity (Resolution) | LOD (ppm) | LOQ (ppm) |
|---|---|---|---|---|---|---|---|---|
| VB | 0 | 2.08 | 2.04 | 98.1 | 1.76 | - | 0.040 | 0.135 |
| 2C1P | 0 | 2.18 | 2.00 | 91.7 | 1.25 | 1.1 | 0.004 | 0.014 |
| 2CP | 0 | 2.30 | 2.17 | 94.3 | 1.64 | 4.2 | 0.013 | 0.042 |
| BE | 0 | 2.16 | 1.67 | 77.3 | 1.77 | 3.7 | 0.010 | 0.033 |
| 2BP | 0 | 2.13 | 2.04 | 95.8 | 0.95 | 14.6 | 0.025 | 0.084 |
| 3C2M1P | 0 | 2.19 | 1.83 | 83.6 | 1.02 | 16.7 | 0.018 | 0.060 |
| 12DCE | 0 | 2.13 | 2.22 | 104.2 | 2.94 | 14.2 | 0.016 | 0.054 |
| 2BB | 0 | 2.17 | 1.75 | 80.6 | 1.63 | 12.7 | 0.005 | 0.019 |
| 12DCP | 0 | 2.12 | 1.83 | 86.3 | 4.26 | 9.7 | 0.023 | 0.081 |
| 4B1B | 0 | 2.09 | 1.61 | 77.0 | 0.29 | 2.8 | 0.011 | 0.038 |
| 1BB | 0 | 2.26 | 1.89 | 83.6 | 0.75 | 8.3 | 0.009 | 0.033 |
| 1B2CE | 0 | 2.19 | 2.09 | 95.4 | 3.59 | 8.4 | 0.031 | 0.205 |
| 11DBE | 0 | 2.13 | 2.06 | 96.7 | 2.59 | 6.0 | 0.029 | 0.098 |
| 3I1P | 0 | 5.24 | 5.46 | 104.2 | 1.13 | - | 0.007 | 0.023 |
| 12DBP | 0 | 5.32 | 5.15 | 96.8 | 1.81 | 26.7 | 0.005 | 0.018 |
| 1B3CP | 0 | 5.39 | 5.16 | 95.7 | 3.87 | 6.2 | 0.012 | 0.040 |
| DIM | 0 | 5.25 | 5.32 | 101.3 | 1.89 | 14.4 | 0.004 | 0.014 |
| 13DBP | 0 | 5.65 | 5.58 | 98.8 | 1.09 | 12.8 | 0.014 | 0.048 |
| 14DBB | 0 | 5.16 | 5.49 | 106.4 | 2.05 | 69.0 | 0.021 | 0.071 |

**Table S9. Applicability of the method on the API Amlodipine camsylate (Cas No. 652969-01-2).**

| | Present amount in API (ppm, P) | Theoretical Spiked amount (ppm, T) | Actual detection Amount (ppm, A) | Recovery ((A-P) / T * 100) (%) | Precision (%RSD) | Specificity (Resolution) | LOD (ppm) | LOQ (ppm) |
|---|---|---|---|---|---|---|---|---|
| VB | 0 | 2.08 | 1.99 | 95.7 | 1.46 | - | 0.044 | 0.147 |
| 2C1P | 0 | 2.18 | 2.12 | 97.2 | 0.74 | 1.1 | 0.004 | 0.014 |
| 2CP | 0 | 2.30 | 2.21 | 96.1 | 1.29 | 4.2 | 0.013 | 0.042 |
| BE | 0 | 2.16 | 2.15 | 99.5 | 1.49 | 3.7 | 0.011 | 0.036 |
| 2BP | 0 | 2.13 | 1.97 | 92.5 | 2.07 | 14.4 | 0.029 | 0.097 |
| 3C2M1P | 0 | 2.19 | 2.01 | 91.8 | 2.15 | 16.6 | 0.016 | 0.054 |
| 12DCE | 0 | 2.13 | 2.03 | 95.3 | 1.83 | 17.7 | 0.017 | 0.058 |
| 2BB | 0 | 2.17 | 1.98 | 91.2 | 2.42 | 12.4 | 0.004 | 0.015 |
| 12DCP | 0 | 2.12 | 1.90 | 89.6 | 13.09 | 9.6 | 0.02 | 0.067 |
| 4B1B | 0 | 2.09 | 1.85 | 88.5 | 4.27 | 2.8 | 0.008 | 0.028 |
| 1BB | 0 | 2.26 | 2.18 | 96.5 | 2.47 | 8.4 | 0.008 | 0.026 |
| 1B2CE | 0 | 2.19 | 2.04 | 93.2 | 0.85 | 8.4 | 0.027 | 0.09 |
| 11DBE | 0 | 2.13 | 1.89 | 88.7 | 3.8 | 6.1 | 0.030 | 0.100 |
| 3I1P | 0 | 5.24 | 6.61 | 126.1 | 2.82 | - | 0.005 | 0.018 |
| 12DBP | 0 | 5.32 | 6.24 | 117.3 | 0.73 | 26.5 | 0.004 | 0.014 |
| 1B3CP | 0 | 5.39 | 6.20 | 115.0 | 1.78 | 6.1 | 0.009 | 0.030 |
| DIM | 0 | 5.25 | 5.36 | 102.1 | 1.23 | 14.7 | 0.004 | 0.013 |
| 13DBP | 0 | 5.65 | 6.36 | 112.6 | 2.5 | 12.9 | 0.012 | 0.038 |
| 14DBB | 0 | 5.16 | 5.79 | 112.2 | 3.75 | 66.6 | 0.017 | 0.056 |

**Table S10. Applicability of the method on the API Ezetimibe (Cas No. 163222-33-1).**

| | Present amount in API (ppm, P) | Theoretical Spiked amount (ppm, T) | Actual detection Amount (ppm, A) | Recovery ((A-P) / T * 100) (%) | Precision (%RSD) | Specificity (Resolution) | LOD (ppm) | LOQ (ppm) |
|---|---|---|---|---|---|---|---|---|
| VB | 0 | 2.08 | 1.97 | 94.7 | 2.23 | - | 0.048 | 0.161 |
| 2C1P | 0 | 2.18 | 2.07 | 95.0 | 0.39 | 1.1 | 0.005 | 0.016 |
| 2CP | 0 | 2.30 | 2.14 | 93.0 | 0.74 | 4.2 | 0.014 | 0.047 |
| BE | 0 | 2.16 | 2.04 | 94.4 | 1.03 | 3.8 | 0.012 | 0.041 |
| 2BP | 0 | 2.13 | 1.90 | 89.2 | 1.31 | 14.5 | 0.032 | 0.106 |
| 3C2M1P | 0 | 2.19 | 1.90 | 86.8 | 1.30 | 16.7 | 0.019 | 0.062 |
| 12DCE | 0 | 2.13 | 2.03 | 95.3 | 1.67 | 8.6 | 0.019 | 0.062 |
| 2BB | 0 | 2.17 | 1.85 | 85.3 | 1.37 | 12.4 | 0.005 | 0.017 |
| 12DCP | 0 | 2.12 | 1.81 | 85.4 | 1.71 | 9.7 | 0.022 | 0.072 |
| 4B1B | 0 | 2.09 | 1.71 | 81.8 | 1.3 | 2.9 | 0.01 | 0.033 |
| 1BB | 0 | 2.26 | 2.07 | 91.6 | 1.46 | 8.3 | 0.009 | 0.029 |
| 1B2CE | 0 | 2.19 | 1.96 | 89.5 | 2.00 | 8.4 | 0.030 | 0.098 |
| 11DBE | 0 | 2.13 | 1.87 | 87.8 | 1.56 | 6.0 | 0.033 | 0.109 |
| 3I1P | 0 | 5.24 | 5.93 | 113.2 | 0.83 | - | 0.005 | 0.016 |
| 12DBP | 0 | 5.32 | 5.24 | 98.5 | 1.19 | 25.1 | 0.004 | 0.012 |
| 1B3CP | 0 | 5.39 | 5.36 | 99.4 | 0.63 | 6.1 | 0.008 | 0.026 |
| DIM | 0 | 5.25 | 5.33 | 101.5 | 2.16 | 14.5 | 0.004 | 0.012 |
| 13DBP | 0 | 5.65 | 5.82 | 103.0 | 1.58 | 12.9 | 0.010 | 0.033 |
| 14DBB | 0 | 5.16 | 6.07 | 117.6 | 4.92 | 67.8 | 0.014 | 0.046 |
